# Hepatocyte-specific perturbation of NAD^+^ biosynthetic pathways in mice induces reversible nonalcoholic steatohepatitis–like phenotypes

**DOI:** 10.1016/j.jbc.2021.101388

**Published:** 2021-11-08

**Authors:** Morten Dall, Anna S. Hassing, Lili Niu, Thomas S. Nielsen, Lars R. Ingerslev, Karolina Sulek, Samuel A.J. Trammell, Matthew P. Gillum, Romain Barrès, Steen Larsen, Steen S. Poulsen, Matthias Mann, Cathrine Ørskov, Jonas T. Treebak

**Affiliations:** 1 Novo Nordisk Foundation Center for Basic Metabolic Research, University of Copenhagen, Copenhagen, Denmark; 2 Novo Nordisk Foundation Center for Protein Research, University of Copenhagen, Copenhagen, Denmark; 3 Department of Biomedical Sciences, University of Copenhagen, Copenhagen, Denmark; 4 Department of Biomedical Sciences, Xlab, Center for Healthy Aging, University of Copenhagen, Copenhagen, Denmark; 5 Clinical Research Centre, Medical University of Bialystok, Bialystok, Poland; 6 Department of Proteomics and Signal Transduction, Max Planck Institute of Biochemistry, Martinsried, Germany

**Keywords:** NAD, NAD^+^ biosynthesis, NAMPT, mitochondria, fibrosis, hepatocyte, AGC, automatic gain control, ALT, alanine aminotransferase, AR, Amplex Red, BSA, bovine serum albumin, CBD, common bile duct, CK-19, cytokeratin-19, DIA, data-independent acquisition, DMEM, Dulbecco's modified Eagle's medium, ECAR, extracellular acidification rate, FBD, fortified breeding diet, FCCP, carbonyl cyanide p-trifluoromethoxyphenylhydrazone, FDR, false discovery rate, GO, Gene Ontology, HNKO, hepatocyte-specific *Nampt* knockout, HRP, horseradish peroxidase, MCD, methionine and choline-free high-fat diet, NA, nicotinic acid, NAFLD, nonalcoholic fatty liver disease, NAMPT, nicotinamide phosphoribosyltransferase, NASH, nonalcoholic steatohepatitis, NMN, nicotinamide mononucleotide, NMNAT, nicotinamide mononucleotide adenylyltransferase, NR, nicotinamide riboside, OCR, oxygen consumption rate, PD, purified control diet, PFA, paraformaldehyde, ROS, reactive oxygen species, SBD, standard breeding diet, SDH, succinate dehydrogenase, SMD, standard maintenance diet, SOD, superoxide dismutase

## Abstract

Nicotinamide phosphoribosyltransferase (NAMPT) converts nicotinamide to NAD^+^. As low hepatic NAD^+^ levels have been linked to the development of nonalcoholic fatty liver disease, we hypothesized that ablation of hepatic *Nampt* would affect susceptibility to liver injury in response to diet-induced metabolic stress. Following 3 weeks on a low-methionine and choline-free 60% high-fat diet, hepatocyte-specific *Nampt* knockout (HNKO) mice accumulated less triglyceride than WT littermates but had increased histological scores for liver inflammation, necrosis, and fibrosis. Surprisingly, liver injury was also observed in HNKO mice on the purified control diet. This HNKO phenotype was associated with decreased abundance of mitochondrial proteins, especially proteins involved in oxidoreductase activity. High-resolution respirometry revealed lower respiratory capacity in purified control diet–fed HNKO liver. In addition, fibrotic area in HNKO liver sections correlated negatively with hepatic NAD^+^, and liver injury was prevented by supplementation with NAD^+^ precursors nicotinamide riboside and nicotinic acid. MS-based proteomic analysis revealed that nicotinamide riboside supplementation rescued hepatic levels of oxidoreductase and OXPHOS proteins. Finally, single-nucleus RNA-Seq showed that transcriptional changes in the HNKO liver mainly occurred in hepatocytes, and changes in the hepatocyte transcriptome were associated with liver necrosis. In conclusion, HNKO livers have reduced respiratory capacity, decreased abundance of mitochondrial proteins, and are susceptible to fibrosis because of low NAD^+^ levels. Our data suggest a critical threshold level of hepatic NAD^+^ that determines the predisposition to liver injury and supports that NAD^+^ precursor supplementation can prevent liver injury and nonalcoholic fatty liver disease progression.

Nonalcoholic fatty liver disease (NAFLD) is a spectrum of diseases ranging from simple steatosis to nonalcoholic steatohepatitis (NASH). It is a major challenge for global health care (1), and as the number of patients increases because of the ongoing obesity pandemic, we need to understand the molecular events that occur from steatosis to steatohepatitis to prevent progression to end-stage liver diseases. Hepatic NAD^+^ levels decrease in livers of obese rodents (2, 3, 4, 5, 6, 7), suggesting NAD^+^ metabolism is a potential target to prevent NAFLD progression. Treatment with NAD^+^ precursors such as nicotinamide riboside (NR) resolves steatosis and diet-induced liver damage in rodents (2, 6, 8), and although more studies are needed, clinical evidence is in line with these observations (9). Thus, improving liver NAD^+^ metabolism may have beneficial effects on liver health in humans.

The majority of the NAD^+^ pool in the liver is derived from tryptophan *via* the *de novo* synthesis pathway and from nicotinamide (10). Nicotinamide phosphoribosyltransferase (NAMPT) converts nicotinamide and phosphoribosyl pyrophosphate to nicotinamide mononucleotide (NMN) and pyrophosphate (11). The NMN adenylyltransferases (NMNAT1–3) then catalyze the condensation of NMN with ATP to generate NAD^+^ (12). NMN can also be generated from NR through phosphorylation by NR kinases 1 and 2 (13), and nicotinic acid (NA) can be converted to NAD^+^ through the Preiss–Handler pathway (14).

A recent study showed that decreased *NAMPT* expression is associated with NASH in humans (15). To investigate the causal relationship between impaired NAD^+^ metabolism and NAFLD, we generated hepatocyte-specific *Nampt* knockout (HNKO) mice (16). We observed that HNKO mice have 50% reduced liver NAD^+^ content, but this did not affect hepatic fatty acid oxidation or respiratory capacity. This was unexpected, as a previous study reported that knockout of *Nampt* impaired fatty acid oxidation and decreased mitochondrial oxygen consumption (17). Other studies suggest that manipulation of NAMPT renders the liver more susceptible toward hepatic lipid accumulation, as both treatment with the NAMPT inhibitor FK866 and overexpression of a dominant-negative *Nampt* mutation induces susceptibility to steatosis development (6, 18). Because an inducible knockout of *Nampt* was shown to impair liver regeneration (19), we hypothesized that HNKO mice would be more likely to develop liver injury if the liver was more severely challenged.

To determine whether HNKO mice had increased susceptibility to develop components of NASH, we challenged HNKO mice and WT littermates with a low-methionine, choline-free 60% high-fat diet (MCD) and evaluated the effects on liver function and metabolic parameters. Collectively, our data suggest the presence of a specific threshold for hepatic NAD^+^ levels that determines the predisposition for developing liver injury. Our data also suggest a zonation pattern in liver injury induced by knockout of *Nampt*, suggesting a spatial requirement for NAD^+^ levels across the liver lobule. In addition, liver injuries induced by subthreshold NAD^+^ levels were found to be associated with a decreased abundance of NAD^+^-dependent oxidoreductases and impaired maximal respiratory capacity. Key features of the liver phenotype were reversible when liver NAD^+^ levels were restored through supplementation with NAD^+^ precursors. Our findings may have implications for liver health in humans.

## Results

### HNKO mice have increased susceptibility to develop liver fibrosis

**Figure 1:**
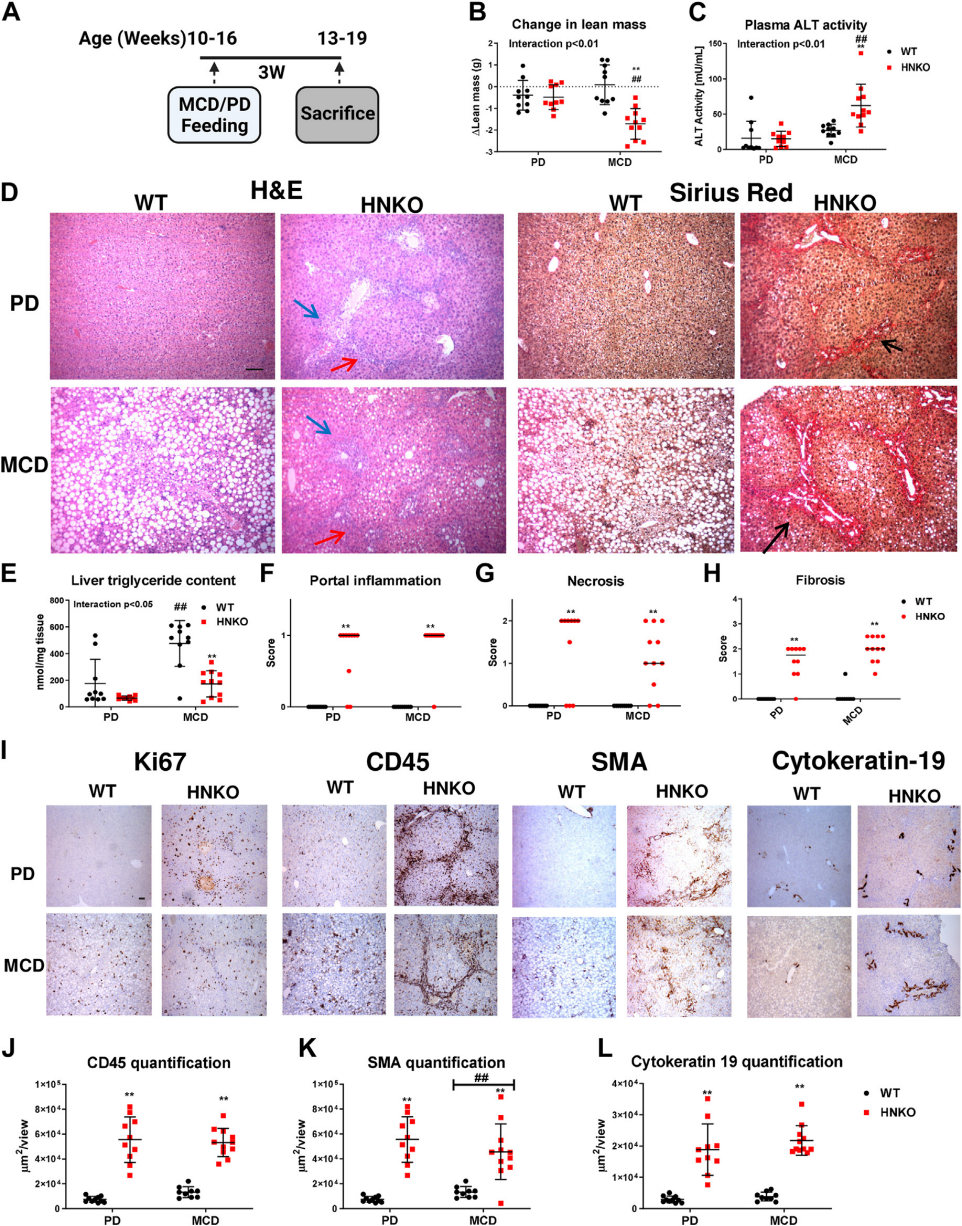
**HNKO mice are more susceptible toward liver damage from MCD diet.** When mice were fed an low MCD for 3 weeks, WT mice accumulated high amounts of triglyceride in the liver, whereas HNKO developed fibrosis in the portal area, focal necrosis, bile duct proliferation, and inflammation. Liver injury was also observed in HNKO mice fed a purified control diet (PD). *A*, experimental setup. HNKO mice and WT littermates were fed an MCD diet or a PD for 3 weeks. *B*, change in lean mass. *C*, plasma ALT activity scores. *D*, H&E-stained and Sirius red-stained liver sections. *E*, hepatic triglyceride quantification and (*F*) portal inflammation scores, (*G*) necrosis scores and (*H*) fibrosis scores. *I*, representative images and quantifications of staining for Ki67, (*J*) CD45, (*K*) smooth muscle actin (SMA), and (*L*) cytokeratin-19. n = 10 to 11. ∗/∗∗ indicate effects of genotype, *p* < 0.05/0.01, respectively. ^#^/^##^ indicate effects of diet, *p* < 0.05/0.01, respectively. Scoring data were analyzed with Kruskal–Wallis test, and mean ranks between groups were compared while correcting for FDR using Benjamini, Krieger, and Yekutieli two-stage step-up method. Parametric data were analyzed with two-way ANOVA with Sidak's multiple comparison post hoc test. *Blue arrow* indicates focal necrosis. *Red arrow* indicates portal inflammation. *Black arrow* indicates fibrosis. The scale bar represents 100 μm. ALT, alanine aminotransferase; FDR, false discovery rate; HNKO, hepatocyte-specific *Nampt* knockout; MCD, methionine and choline-free high-fat diet.

To determine if lack of *Nampt* would induce liver injury, we challenged female HNKO mice with an MCD diet for 3 weeks (Fig. 1*A*). Lean mass and plasma alanine aminotransferase (ALT) activity were unaffected in WT mice, but HNKO mice lost 1.5 g of lean mass (Fig. 1*B*; *p* < 0.01) and had a three-fold increase in plasma ALT activity in response to the MCD diet (Fig. 1*C*; *p* < 0.01). This indicated liver injury in HNKO mice. Body weight (BW) and fat mass were not affected by MCD feeding (Fig. S1*A* and *B*). Hepatic NAD^+^ content was ∼66% lower in purified control diet (PD)-fed HNKO mice compared with WT littermates, but no additional decrease following MCD diet feeding was observed (Fig. S1*C*; *p* < 0.01). NADP^+^ levels were ∼20% lower in PD-fed HNKO mice compared with WT (*p* < 0.01), and a decrease in NADP^+^ was observed for both genotypes following MCD diet feeding (Fig. S1*D*; *p* < 0.05). NADPH levels were lower in PD-fed HNKO mice compared with WT littermates, and MCD feeding decreased NADPH levels in WT mice but not HNKO mice (Fig. S1*E*; *p* < 0.05). HNKO mice developed periportal fibrosis, immune cell infiltration, bile duct proliferation, and focal necrosis (Fig. 1*D*) and showed lower accumulation of hepatic triglyceride from the MCD diet challenge than WT mice (Fig. 1*E*). Remarkably, portal inflammation, necrosis, and fibrosis were also observed in HNKO mice on the PD, and scores were not significantly different between the MCD group and the control group (Fig. 1, *F*–*H*). However, there was no decrease in lean mass and no increase in plasma ALT activity in the PD HNKO group. HNKO livers appeared to have more proliferating cells (Ki-67, Fig. 1*I*) and had a significant increase in leukocyte infiltration in the portal areas for both diet groups (CD45, Fig. 1, *I* and *J*). Myofibroblast activation and bile duct proliferation was observed in HNKO mice regardless of diet (smooth muscle actin (SMA) and cytokeratin-19 [CK-19] Fig. 1, *K* and *L*). Overall, HNKO mice were more susceptible to damage from the MCD diet but developed liver fibrosis, inflammation, and bile duct proliferation even on the control diet.

### Liver injury in PD-fed HNKO is associated with increased abundance of NAFLD-associated plasma proteins

**Figure 2:**
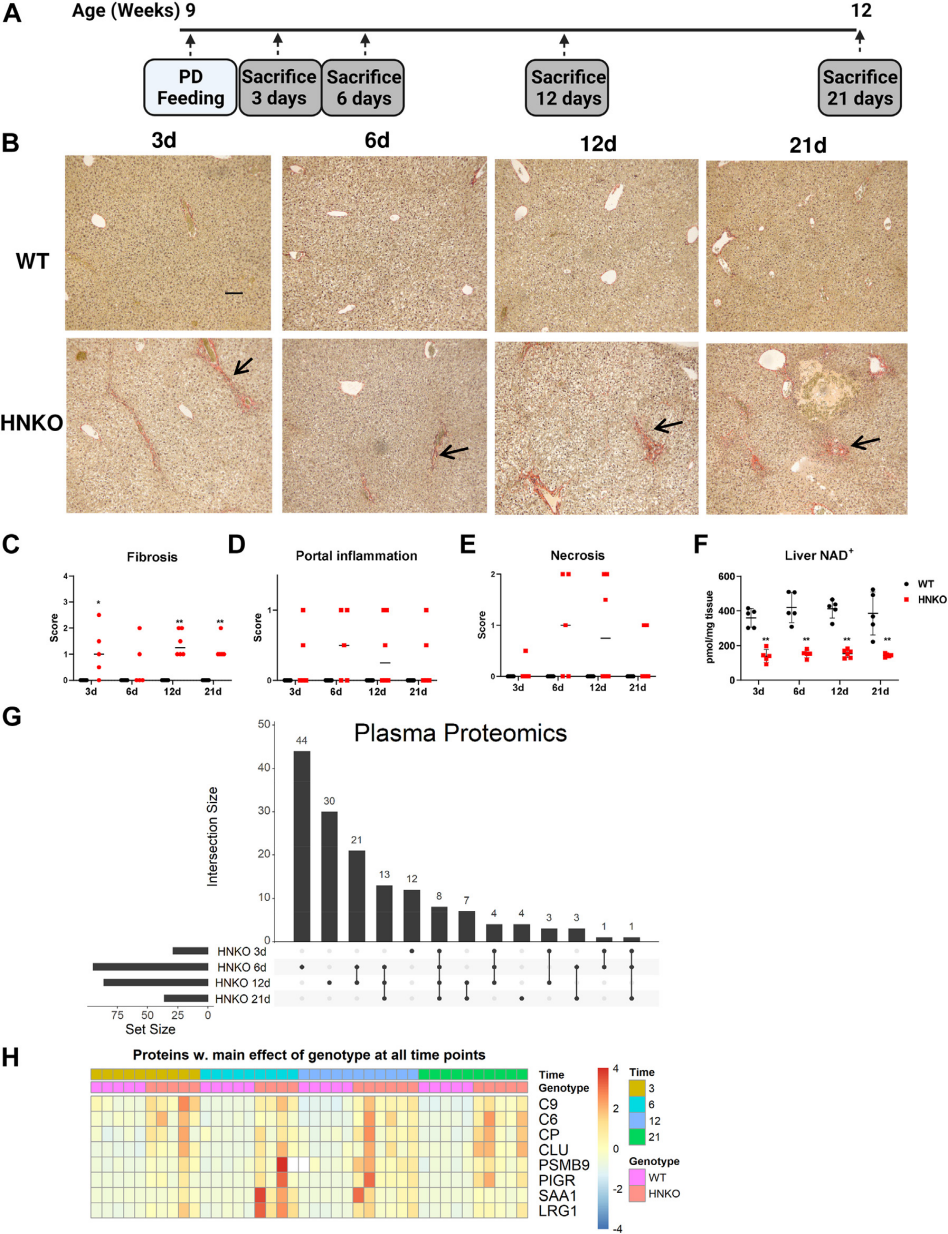
**The HNKO phenotype is present after 3 days of PD feeding.** To determine whether the HNKO phenotype was induced by PD feeding, HNKO and WT mice were fed PD from 3 to 21 days. This revealed that fibrosis was present after 3 days of PD feeding. *A*, experimental setup. *B*, representative Sirius red-stained liver sections and (*C–E*) histology scores. *F*, liver NAD^+^ levels. *G*, upset plot showing the number of plasma proteins with a significantly altered abundance between genotypes at the four time points. *H*, heatmap displaying plasma content of eight proteins with a significantly different abundance between genotypes at all investigated time points. n = 5 to 6. ∗/∗∗ indicates effects of genotype, *p* < 0.05/0.01, respectively. Scoring data were analyzed with Kruskal–Wallis test, and mean ranks between groups were compared while correcting for FDR using Benjamini, Krieger, and Yekutieli two-stage step-up method. Parametric data were analyzed with two-way ANOVA with Sidak's multiple comparison post hoc test. Differentially abundant plasma proteins were identified using the R package Limma. *Black arrow* indicates fibrosis. The scale bar represents 100 μm. FDR, false discovery rate; HNKO, hepatocyte-specific *Nampt* knockout; PD, purified control diet.

To determine temporal patterns in the development of liver injury in PD-fed HNKO mice, 9-week-old WT and HNKO mice were fed PD for 3, 6, 12, and 21 days (Fig. 2*A*). Fibrosis was present after 3 days (Fig. 2, *B* and *C*; *p* < 0.05), although the response was heterogeneous until the 12-day time point where all HNKO mice exhibited a positive fibrosis score (*p* < 0.01). Portal inflammation and necrosis varied between HNKO mice across the time course (Fig. 2, *D* and *E*). Liver NAD^+^ levels were decreased by 66% in HNKO mice and did not change over time (Fig. 2*F*; *p* < 0.01). In both humans and mice, NAFLD and NASH affect the plasma proteome, and content of plasma biomarkers may predict NAFLD/NASH development (20). To investigate whether the plasma biomarker profile of HNKO mice was similar to other models of NAFLD and NASH, we analyzed the plasma proteome using MS. We identified that the highest number of differentially abundant proteins between genotypes were observed at day 6 and 12 (Fig. 2*G*, File S1). Eight proteins were differentially abundant in plasma between genotypes at all time points and were all increased in HNKO plasma compared with plasma from WT mice (Fig. 2*H*). Increased serum amyloid A1 abundance was previously reported in mice fed a high-fat diet for 6 months (20), and polymeric immunoglobulin receptor was identified as a candidate plasma biomarker for human NAFLD and cirrhosis (20). These data indicate the presence of fibrosis in HNKO mice already after 3 days on the PD, and they confirm the relevance of previously identified biomarker proteins for NAFLD.

### Liver injury in HNKO is associated with major changes to hepatic transcriptome and proteome

**Figure 3:**
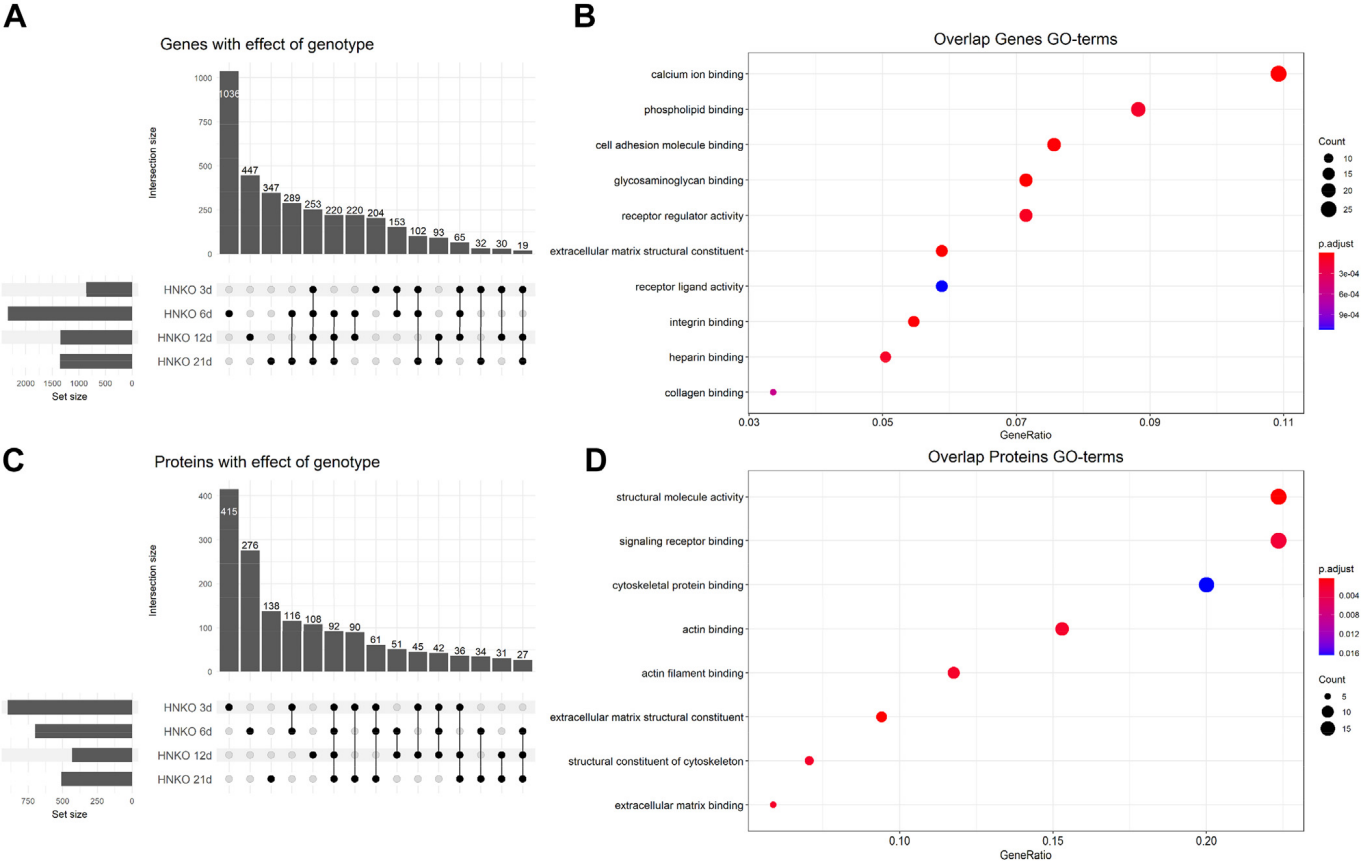
**mRNA expression and protein abundance of hepatic genes/proteins supports ongoing tissue remodeling and extracellular matrix deposition in HNKO mice.** To determine the changes associated with the HNKO phenotype, we sequenced the HNKO transcriptome and quantified the liver proteome. The analysis revealed that damage was occurring throughout the 21 days, and that the HNKO phenotype was associated with an altered abundance of proteins involved in oxidation–reduction processes. *A*, upset plot showing overlap between genes with a significantly altered expression between genotypes at the four time points. *B*, Gene Ontology (GO) enrichment analysis of genes with a differential expression between genotypes at all four time points. *C*, upset plot showing overlap between proteins with a significantly altered abundance between genotypes at the four time points. *D*, GO enrichment analysis of proteins with a significantly altered abundance between genotypes at the four investigated time points. RNA-Seq data were analyzed with the R package EdgeR for differential expression testing, using a model of the form *expression ∼ Group*. Significantly enriched GO terms were identified using the R-package ClusterProfiler. Differential abundance of hepatic proteins was tested using the R package Limma. HNKO, hepatocyte-specific *Nampt* knockout.

To determine potentially underlying mechanisms for PD-associated liver injury, we assessed time course dependent gene expression changes using RNA-Seq. Similarly to the observed changes in the plasma proteome, the largest number of differentially expressed genes in the liver between HNKO and WT was observed at day 6, where more than 2000 genes were differentially expressed (Fig. 3*A*, File S2). About 239 genes were found to be differentially expressed at all time points, and Gene Ontology (GO) enrichment analysis for “molecular function” revealed enrichment of terms such as *calcium ion binding, cell adhesion molecule binding*, and *extracellular matrix structural constituent* for this group, suggesting the presence of organ remodeling and extracellular matrix deposition at all investigated time points (Fig. 3*B*). We noted an increased expression of several markers for oval cells and cholangiocytes (21), including *Epcam, Ncam1, Spp1, Sox9*, and *Krt7* in HNKO livers throughout the time course (Fig. S2, *A*–*E*). Similarly, we observed a decreased expression of albumin after 6 days (Fig. S2*F*). It has been suggested that cholangiocytes and oval cells can contribute to liver regeneration through transdifferentiation when hepatocyte proliferation is impaired (21). Hence, the increased expression of these markers suggests that ductular reaction and fibrosis may be a compensatory event in HNKO livers to support liver regeneration. To investigate whether the major transcriptional changes manifested as changes to the proteome, we quantified the proteome of HNKO and WT livers from the same samples. In contrast to the transcriptomics analysis, the largest number of differentially abundant proteins was observed at day 3 (Fig. 3*C* and File S3). About 92 proteins were differentially abundant at all four time points, and “molecular function” GO terms enriched for these proteins included *structural molecule activity, extracellular matrix structural constituent*, and *extracellular matrix binding* (Fig. 3*D*). Thus, these proteomics data confirm a continuous damage/regeneration response in HNKO mice fed a PD.

### Liver injury depends on dietary NAD^+^ precursors, and fibrosis in HNKO mice correlates with hepatic NAD^+^ content

**Figure 4:**
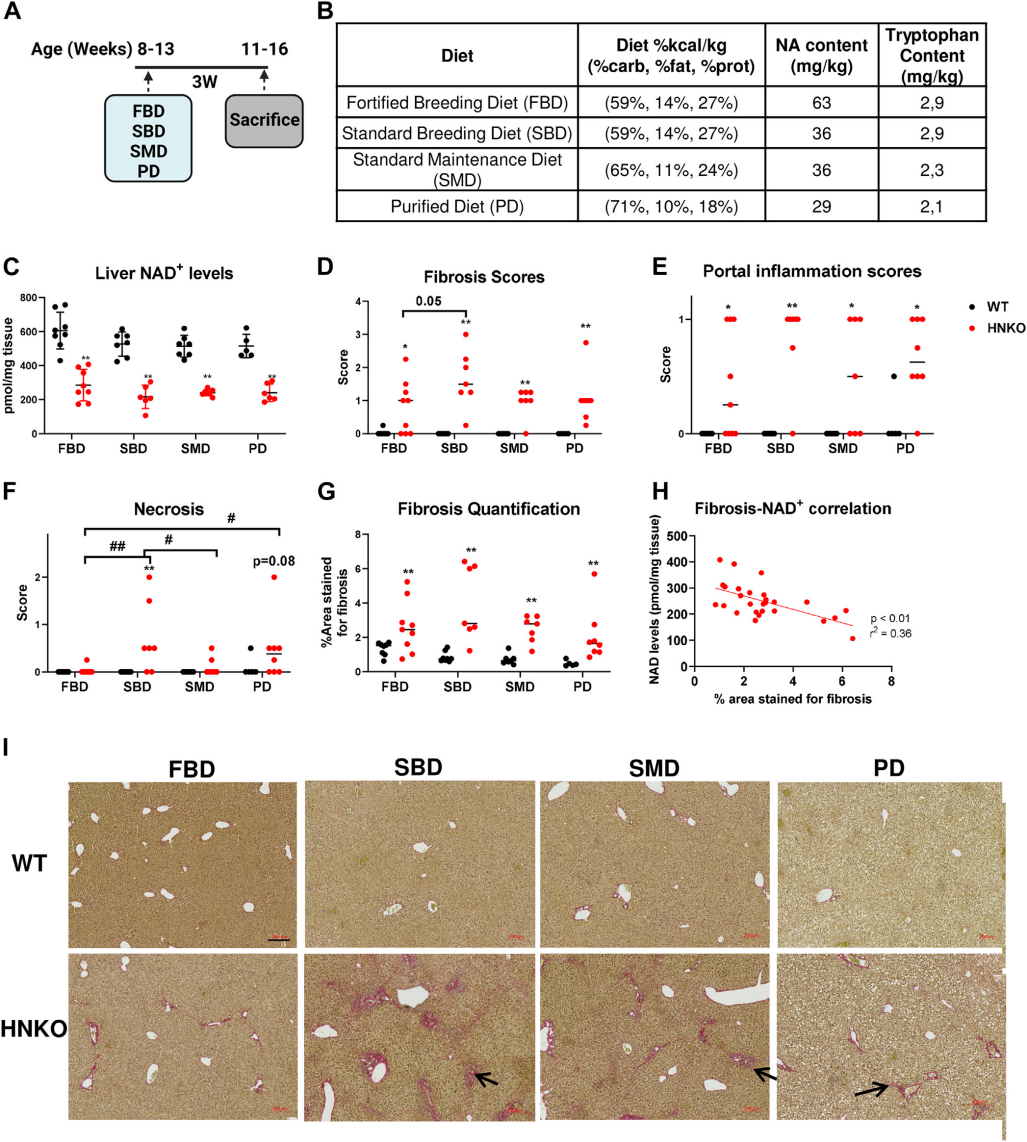
**Hepatic NAD**^**+**^**content correlates with fibrosis severity in HNKO mice.** To test whether differences in diet composition or nicotinic acid content affected fibrosis development, three different chow diets + PD were tested in HNKO mice and WT littermates. *A*, experimental setup. *B*, the table showing composition of the four tested diets. *C*, liver NAD^+^ content. *D*, fibrosis scores, (*E*) portal inflammation scores, and (*F*) necrosis scores. *H*, quantification of areas stained for fibrosis on sections. *I*, correlation between sizes of area stained for fibrosis and liver NAD^+^ levels in HNKO. n = 5 to 8. ∗/∗∗ indicate effects of genotype, *p* < 0.05/0.01, respectively. ^#^/^##^ indicate effects of diet, *p* < 0.05/0.01, respectively. The scale bar represents 200 μm. HNKO, hepatocyte-specific *Nampt* knockout; PD, purified control diet.

Because fibrosis was observed after only 3 days of PD feeding, it was likely that fibrosis was present before PD feeding. To test this idea and further evaluate whether the micronutrient and macronutrient composition affected fibrosis development in HNKO mice, we fed 8 to 13-week-old HNKO and WT mice four different diets with a different NAD^+^ precursor content for 3 weeks (Fig. 4*A*). We tested PD, a fortified breeding diet (FBD; the standard chow in our animal facility), a standard breeding diet (SBD), and a standard maintenance diet (SMD). Fortified diets have a higher content of vitamins (including NA) compared with standard diets, whereas breeding diets have a higher content of fat and amino acids (including tryptophan) compared with maintenance diets (Fig. 4*B*). All mice started on FBD following weaning and until the beginning of the experiment. We observed a main effect of diet for hepatic NAD^+^ content (Fig. 4*C*; *p* < 0.05). Post hoc testing revealed a tendency toward increased liver NAD^+^ levels for FBD-fed groups compared with other diets, though this was not statistically significant (SBD: *p* = 0.07; SMD: *p* = 0.1; PD: *p* = 0.14). NAD^+^ levels were lower in HNKO liver for all diet groups (Fig. 4*C*, *p* < 0.01). Fibrosis and portal inflammation scores were significantly higher in HNKO mice for all diets, and necrosis scores were significantly increased in SBD-fed HNKO mice compared with SBD-fed WT mice (Fig. 4, *D*–*F* and *I*, *p* < 0.05). We observed a tendency toward lower fibrosis scored in FBD-fed HNKO mice compared with SBD-fed HNKO mice (Fig. 4*D*; *p* = 0.05) and significantly lower necrosis scores in FBD-fed HNKO mice compared with PD-fed and SBD-fed HNKO mice (Fig. 4*F*; *p* < 0.05). Notably, in several FBD-fed HNKO mice, fibrosis was absent, suggesting that the higher NA content protected some HNKO mice from fibrosis development (Fig. 4*D*). In contrast, fibrosis did not seem to be dependent on tryptophan intake, as fibrosis developed in SBD-fed HNKO mice. When fibrotic areas in sections were quantified, we saw that HNKO livers had significantly larger areas stained for fibrosis compared with WT animals across all diet groups (Fig. 4*G*; *p* < 0.01). A significant and negative correlation between stained fibrosis area and hepatic NAD^+^ content was observed in HNKO mice (Fig. 4*H*; Pearson *r*^2^ = 0.36; *p* < 0.01). Collectively, although necrosis in the HNKO livers are dependent on the dietary NAD^+^ precursor content, fibrosis was present irrespective of the diet and correlated negatively with hepatic NAD^+^ content.

### Liver injury in HNKO mice can be attenuated by NR and NA supplementation

**Figure 5:**
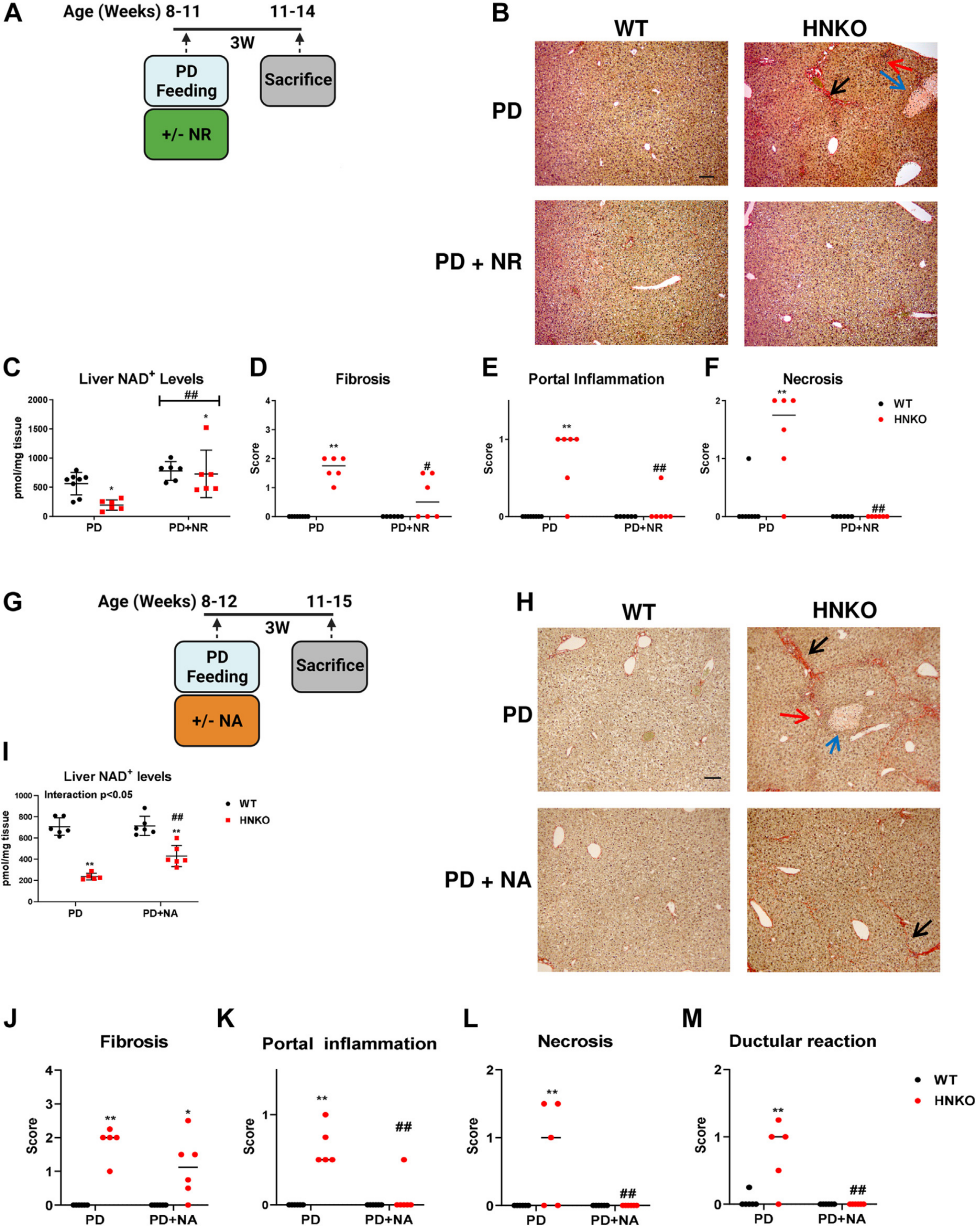
**Liver damage in HNKO mice can be prevented by supplementation with NAD**^**+**^**precursors.** To determine whether the HNKO phenotype could be alleviated by NAD^+^ repletion, we supplied PD-fed HNKO mice with the precursors nicotinamide riboside (NR) and nicotinic acid (NA). Supplementation with the individual precursors was sufficient to attenuate PD-associated liver injury in HNKO mice. *A*, WT and HNKO mice were fed with PD for 3 weeks, receiving either normal drinking water or water containing 1.9 g NR/l . *B*, representative Sirius red images of liver sections are displayed. *C*, hepatic NAD^+^ levels and (*D*) fibrosis scores, (*E*) portal inflammation scores, and (*F*) necrosis scores; n = 6. *G*, WT and HNKO mice were fed a PD with increased NA content (PD+NA, 75 mg/kg diet) or normal PD (29 mg/kg diet) for 3 weeks. *H*, representative Sirius red images are displayed. *I*, hepatic NAD^+^ content. *J*, fibrosis scores, (*K*) portal inflammation scores, (*L*) necrosis scores, and (*M*) ductular reaction scores obtained from Sirius red stainings. n = 5 to 6. ∗/∗∗ indicate effects of genotype, *p* < 0.05/0.01, respectively. ^#^/^##^ indicate effects of treatment (NA or NR), *p* < 0.05/0.01, respectively. Scoring data were analyzed with Kruskal–Wallis test, and mean ranks between groups were compared while correcting for FDR using Benjamini, Krieger, and Yekutieli two-stage step-up method. Parametric data were analyzed with two-way ANOVA with Sidak's multiple comparison post hoc test. *Blue arrow* indicates focal necrosis. *Red arrow* indicates portal inflammation. *Black arrow* indicates fibrosis. The scale bar represents 100 μm. FDR, false discovery rate; HNKO, hepatocyte-specific *Nampt* knockout; PD, purified control diet.

Fibrosis in HNKO mice was associated with decreased hepatic NAD^+^ content, and we therefore hypothesized that increasing hepatic NAD^+^ content could alleviate this phenotype. NR and NA are precursors of NAD^+^ that do not depend upon NAMPT for NAD^+^ synthesis. To investigate if fibrosis in HNKO mice could be prevented by NR, HNKO and WT mice were fed PD with or without NR in the drinking water for 3 weeks (Fig. 5, *A* and *B*). Hepatic NAD^+^ content in HNKO mice was three-fold higher with NR (Fig. 5*C*; *p* < 0.01), and they had significantly lower fibrosis scores, portal inflammation scores, and necrosis scores compared with the control group (Fig. 5, *D*–*F*; *p* < 0.05). To test whether supplementation with smaller amounts of NAD^+^ precursor had similar beneficial effects, we fed HNKO mice a PD with an NA content of 75 mg/kg for 3 weeks (Fig. 5, *G* and *H*). The additional supplementation of NA to the PD increased NAD^+^ levels two-fold in HNKO livers (Fig. 5*I*; *p* < 0.01). NA supplementation did not affect fibrosis scores (Fig. 5*J*) but significantly reduced portal inflammation scores (Fig. 5*K*; *p* < 0.01), necrosis scores (Fig. 5*L*; *p* < 0.01) and ductular reaction scores (Fig. 5*M*; *p* < 0.01). Together, these data demonstrate that increasing liver NAD^+^ levels attenuates liver damage in HNKO mice, and they also suggest the presence of a threshold for NAD^+^ under which liver damage occurs in HNKO mice.

### NR attenuates hepatic inflammation PD-fed HNKO mice

**Figure 6:**
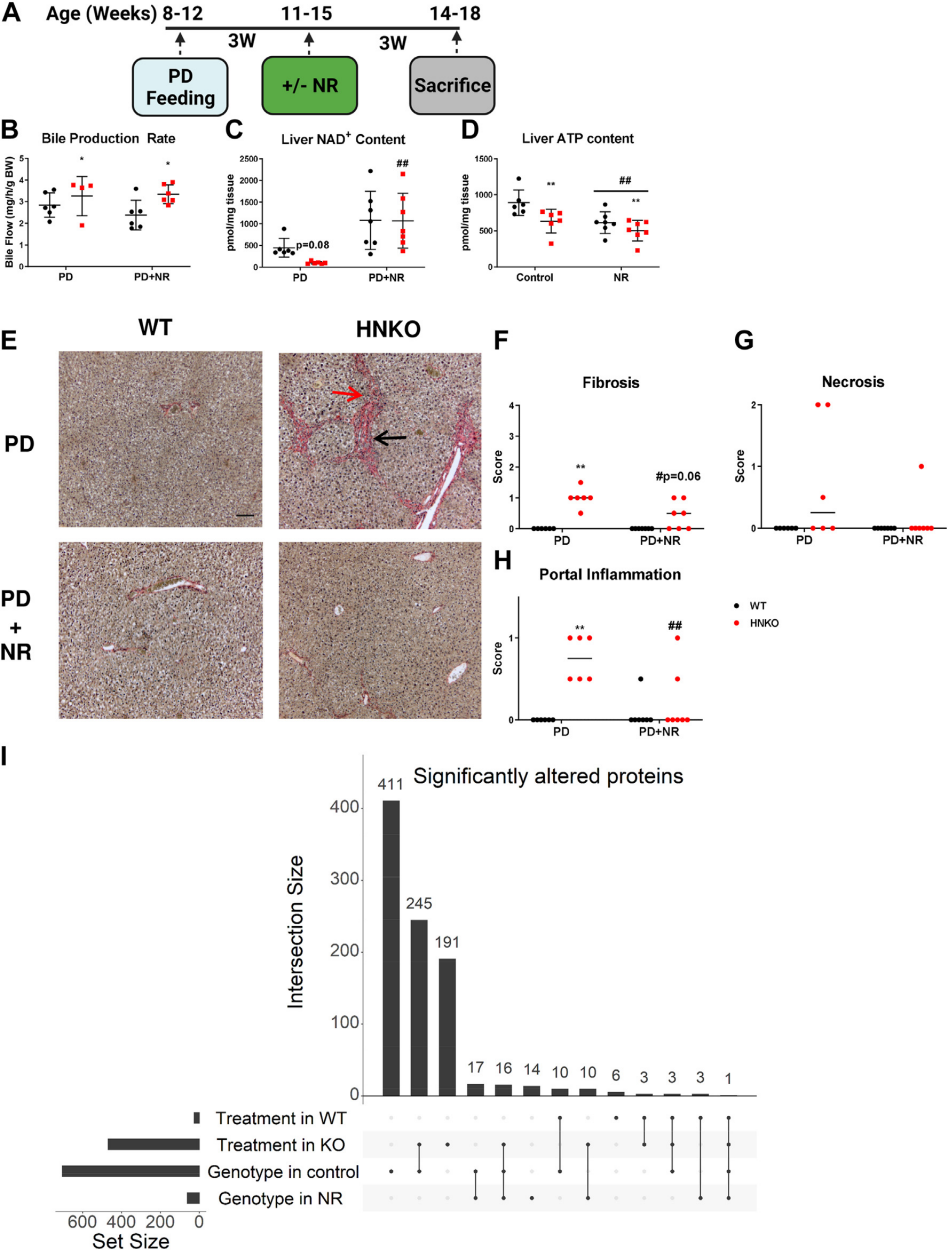
**NR intervention attenuates portal inflammation in HNKO mice.** To determine whether NR treatment could improve the HNKO phenotype when given as an intervention, HNKO and WT mice were fed PD for 6 weeks, receiving drinking water ±1.9 g/l NR for the final 3 weeks. Fibrosis scores were borderline decreased, whereas portal inflammation scores in HNKO mice were significantly improved by NR supplementation. *A*, experimental setup. *B*, bile production rate, (*C*) liver NAD^+^-content, and (*D*) liver ATP content. *E*, representative Sirius red-stained liver sections with (*F*–*H*) histological scores. *I*, upset plot showing proteins with a significantly altered abundance between genotypes or following NR treatment. n = 6 to 7. ∗/∗∗ indicate effects of genotype, *p* < 0.05/0.01, respectively. ^#^/^##^ indicate effects of NR, *p* < 0.05/0.01, respectively. Scoring data were analyzed with Kruskal–Wallis test, and mean ranks between groups were compared while correcting for FDR using Benjamini, Krieger, and Yekutieli two-stage step-up method. Parametric data were analyzed with two-way ANOVA with Sidak's multiple comparison post hoc test. Differential abundant proteins were identified through the R package Limma. *Red arrow* indicates portal inflammation. *Black arrow* indicates fibrosis. The scale bar represents 100 μm. FDR, false discovery rate; HNKO, hepatocyte-specific *Nampt* knockout; NR, nicotinamide riboside; PD, purified control diet.

Bile duct–ligated mice present a similar phenotype to HNKO mice with a strong zonation of fibrosis in the portal areas of the lobuli (22). Therefore, we investigated whether hepatic NAD^+^ deficiency was associated with cholestasis by measuring bile flow in HNKO and WT mice. WT and HNKO mice were fed PD for 6 weeks, and half of the mice received NR in their drinking water after the first 3 weeks (Fig. 6*A*). This would show whether NR reverses the phenotype induced by decreased NAD^+^ precursor intake. Interestingly, we found bile flow to be slightly increased in HNKO mice (Fig. 6*B*), possibly because of the enlargened bile ducts. Bile flow was not corrected by NR supplementation suggesting that the HNKO phenotype is not associated with cholestasis. NAD^+^ content in HNKO mice was 75% lower than for WT animals, and NR supplementation caused a 10-fold increase in hepatic NAD^+^ content in HNKO mice (Fig. 6*C*; *p* < 0.01). HNKO livers had a significantly lower content of ATP, which was not corrected by NR (Fig. 6*D*; *p* < 0.01). Rather, NR caused a decrease in hepatic ATP content regardless of genotype (*p* < 0.01). Three of seven HNKO mice had a fibrosis score of 0 following NR treatment (Fig. 6, *E* and *F*; *p* = 0.06). No significant change in necrosis scores was observed (Fig. 6*G*), but portal inflammation was significantly decreased in the NR-supplemented HNKO group (Fig. 6*H*; *p* < 0.01). Thus, liver injury in the HNKO mice is not caused by cholestasis, and NR effectively reduces liver inflammation.

### NR supplementation rescues hepatic abundance of NAD^+^-dependent oxidoreductases

**Figure 7:**
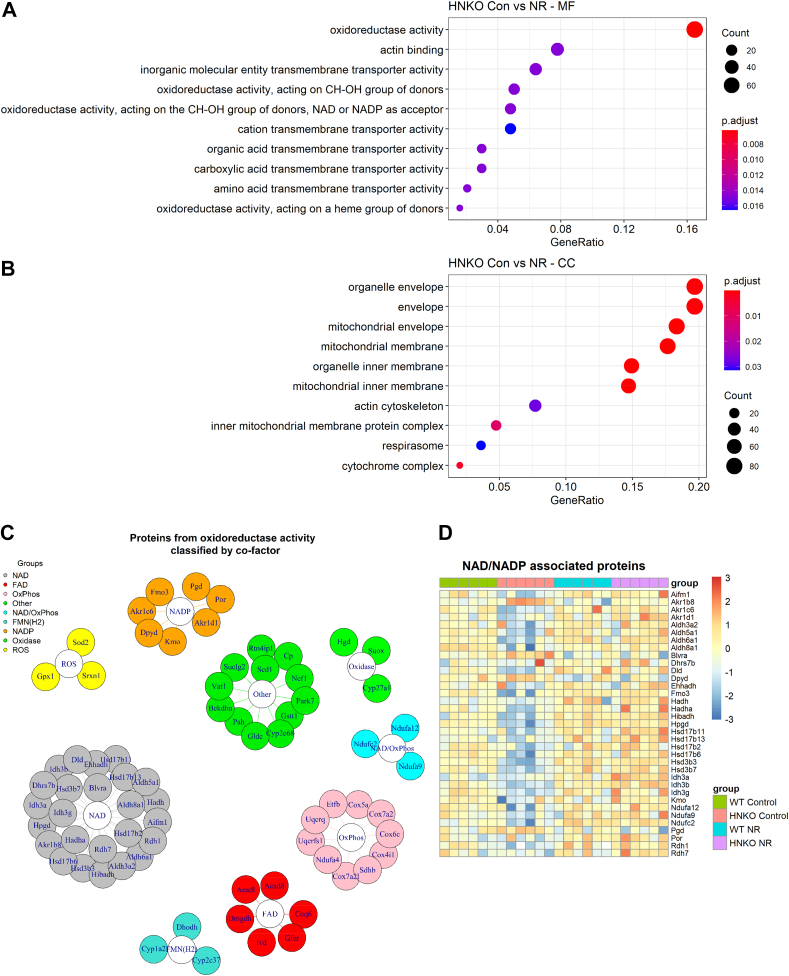
**NR normalizes hepatic abundance of NAD**^**+**^**-dependent oxidoreductase proteins in HNKO mice.** Analysis of the HNKO liver proteome following NR supplementation revealed that NR significantly increased abundance of mitochondrial proteins and oxidoreductases. This observation supports that NR supplementation is associated with improved mitochondrial function in HNKO mice. Gene Ontology enrichment analysis of liver proteins with a differential abundance between HNKO mice ±NR supplementation for (*A*) molecular function (MF) and (*B*) cellular component (CC). *C*, overview of proteins from the *oxidoreductase activity* term with a differential abundance between HNKO mice ±NR supplementation, grouped by cofactor and/or cellular function. Proteins were annotated to a group based on their description in the database UniProt (23). *D*, heatmap showing abundance of proteins from the *oxidoreductase activity* term that uses either NAD^+^, NADH, or NADP^+^ for their reactions. n = 6 to 7. HNKO, hepatocyte-specific *Nampt* knockout; NR, nicotinamide riboside.

Next, we mapped the liver proteome to identify NR-associated changes in HNKO mice. Major differences in protein abundance were observed between WT and HNKO in the control group and between control and NR-treated HNKO mice (Fig. 6*I* and File S4). Remarkably, only few differentially abundant proteins were altered by genotype in the NR groups and by NR in WT mice. This clearly indicates that NR has only minor effects in livers of WT mice but that it reverses the proteome profile in the HNKO liver to resemble that of WT mice. Furthermore, this clearly highlights lack of NAD^+^ as a direct mechanism for the observed phenotype. GO enrichment analyses of proteins with significantly altered abundance between genotypes in the control group were enriched for “molecular function” terms associated with cell motility and fibrosis, such as *cytoskeletal protein binding* and *extracellular matrix structural constituent* (Fig. S3*A*). GO enrichment analysis for “cellular component” showed not only also an enrichment of extracellular matrix–associated terms, such as *extracellular region,* but also of mitochondria-associated terms such as *mitochondrial membrane* and *mitochondrial envelope* (Fig. S3*B*)*.* Hence, the proteomics analysis suggested altered mitochondrial protein content in PD-fed HNKO mice. GO analysis of hepatic proteins with a significantly altered abundance following NR treatment in HNKO mice showed an enrichment of molecular function terms, such as *oxidoreductase activity, actin binding,* and *oxidoreductase activity acting on CH-OH group of donors using NAD or NADP as acceptor* (Fig. 7*A*). We observed a significant enrichment of cellular component terms, including *mitochondrial envelope, mitochondrial membrane,* and *actin cytoskeleton*, suggesting that NR attenuated cell motility and/or cytoskeletal rearrangements, while also affecting mitochondrial protein abundance. To further explore the cellular processes that were affected in HNKO livers, we annotated the proteins from the *oxidoreductase activity* term according to cofactor and/or cellular function, based on entries in the database UniProt (23). This classification revealed that a high number of these proteins use NAD(H) or NADP(H) as cofactors (Fig. 7*C*). A heatmap representation of these proteins showed a clear overall decrease in the abundance of these proteins in livers of HNKO mice, which was normalized by NR treatment (Fig. 7*D*). This was also the case when all differentially abundant proteins from the *oxidoreductase activity* term were plotted in a heatmap (Fig. S4*A*). Hence, NR treatment rescued hepatic NAD^+^-dependent oxidoreductase protein abundance.

### HNKO livers have no excessive production of reactive oxygen species or alterations in mitochondrial content

**Figure 8:**
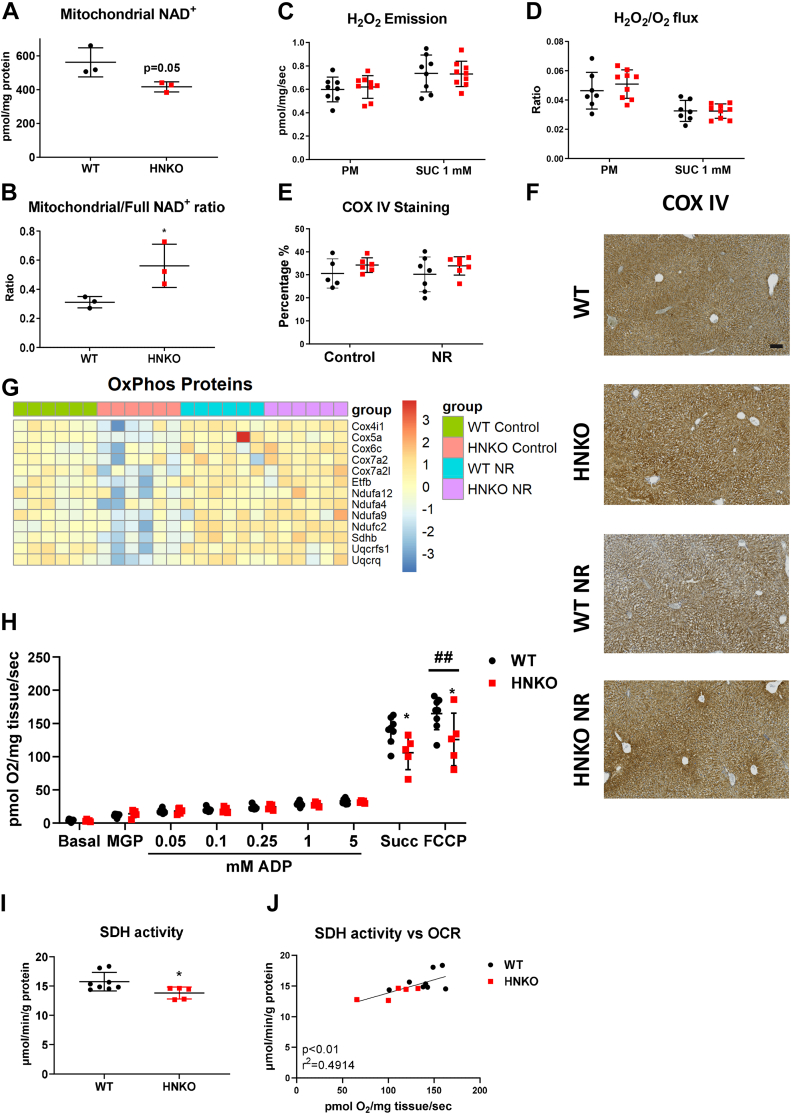
**HNKO livers have reduced maximal respiratory capacity and decreased succinate dehydrogenase (SDH) activity.** High-resolution respirometry revealed that the HNKO phenotype was associated with decreased oxygen consumption following succinate addition, suggesting decreased coupled complex I+II function. HNKO mice had no change in mitochondrial content or in ROS production. *A*, mitochondrial NAD^+^ content in HNKO and WT mice after 3 weeks of PD feeding. *B*, mitochondrial to full NAD^+^ ratio in HNKO and WT liver, n = 3. *C*, H_2_O_2_ emission and (*D*) H_2_O_2_/O_2_ flux from WT and HNKO liver samples following 3 weeks of PD feeding. Emission and ratios were determined after the addition of pyruvate and malate (PM) and succinate (SUC; 1 mM). n = 8. *E*, quantification of COX IV staining in liver sections from the NR supplementation study. n = 6 to 7. *F*, representative images of the COX IV stainings. The scale bar represents 100 μm. *G*, heatmap displaying abundance of OXPHOS proteins with a differential abundance between HNKO mice ±NR. *H*, respirometry data from WT and HNKO liver samples following 3 weeks of PD feeding. After a basal measurement, samples added were malate, glutamate, and pyruvate (MGP), followed by increasing amounts of ADP, followed by succinate, and last FCCP. *I*, SDH activity in liver lysates from the respirometry analysis. *J*, correlation data between succinate-stimulated oxygen consumption and SDH activity. n = 5 to 8. ∗ indicates effects of genotype, *p* < 0.05 and ^#^/^##^ indicates effect of treatment, *p* < 0.05 and *p* < 0.01, respectively. FCCP, carbonyl cyanide p-trifluoromethoxyphenylhydrazone; HNKO, hepatocyte-specific *Nampt* knockout; NR, nicotinamide riboside; PD, purified control diet; ROS, reactive oxygen species.

As NR supplementation altered the abundance of many mitochondrial proteins in HNKO mice, we hypothesized that the HNKO phenotype could be associated with lower mitochondrial content. We observed a tendency toward decreased mitochondrial NAD^+^ levels in HNKO mice fed a PD diet compared with WT littermates (Fig. 8*A*, *p* = 0.05); although the mitochondrial to total NAD^+^ ratio was increased in HNKO mice (Fig. 8*B*; *p* < 0.05). Hence, the decreased abundance of mitochondrial proteins was not associated with severe mitochondrial NAD^+^ depletion. We observed a lower abundance of superoxide dismutase 2 (SOD2) and glutathione peroxidase 1 in HNKO mice (Figs. 7*C* and S4*A*). We therefore hypothesized that HNKO mice could have impaired reactive oxygen species (ROS) handling capabilities. However, basal and succinate-induced hydrogen peroxide (H_2_O_2_) production was not significantly affected in livers from PD-fed HNKO mice (Fig. 8*C*). Similarly, the H_2_O_2_/O_2_ ratio was not changed by genotype in either state, again suggesting no increased ROS production from HNKO livers (Fig. 8*D*). Similarly, we detected no change in protein carbonylation in HNKO livers from the NR experiment, indicating that ROS were not excessively produced (Fig. S4*B*). To investigate whether the HNKO phenotype was associated with an altered content of mitochondria, we quantified mitochondrial content by staining complex IV on liver sections from the NR intervention experiment (Fig. 6*A*). Quantification of stained areas showed no difference between HNKO and WT mice (Fig. 8, *E* and *F*). Similarly, citrate synthase activity was unaltered between groups, suggesting unchanged mitochondrial content in all groups (Fig. S4*C*). Thus, decreased abundance of oxidoreductase proteins in HNKO mice was not associated with excessive ROS production or decreased content of mitochondria.

### HNKO livers have impaired maximal respiratory capacity

We noted that several proteins from the *Oxidoreductase activity* GO term were subunits of OXPHOS complexes (Fig. 7*C*). Most of the differentially abundant OXPHOS proteins had decreased abundance in HNKO mice and were rescued by NR supplementation (Fig. 8*G*). To test whether this altered respiratory capacity, we performed high-resolution respirometry on livers from WT and HNKO mice following 3 weeks of PD feeding. Although no defects in coupled complex I respiration were observed, succinate- and carbonyl cyanide p-trifluoromethoxyphenylhydrazone (FCCP)-induced respiration were 20% lower in HNKO livers (Fig. 8*H*, *p* < 0.05). These data demonstrate that the decreased abundance of OXPHOS proteins in the proteomics analysis was associated with impaired maximal respiratory capacity of complex I+II. The decreased activity of complex I + II could be due to decreased abundance of the complex II subunit succinate dehydrogenase B (SDHB) (Fig. 8*G*). Accordingly, activity of the SDH complex was lower in liver homogenate from PD-fed HNKO mice compared with WT littermates (Fig. 8*I*; *p* < 0.05). Moreover, we observed a correlation between SDH activity and succinate-stimulated oxygen consumption rate (OCR) (Fig. 8*J*; *p* < 0.01; Pearson *r*^2^ = 0.49), suggesting that the lower OCR was caused by decreased complex II activity rather than substrate deficiency. Hence, HNKO livers have decreased mitochondrial respiratory capacity, which is likely a direct effect of decreased complex II activity.

### HNKO livers have altered abundance of proteins involved in NAD^+^ synthesis and consumption

Lack of adaptation in the abundance of proteins involved in NAD^+^ synthesis and/or consumption could contribute to the liver injury phenotype in the HNKO mice. Interestingly, PD-fed HNKO mice had lower levels of proteins involved in NAD^+^ synthesis, including the mitochondrial protein NMNAT3, and several enzymes from the NAD^+^ *de novo* synthesis pathway (Fig. S4*D*). Similarly, abundances of multiple NAD^+^-consuming enzymes were altered, and HNKO livers had a decreased abundance of NADK and the mitochondrial sirtuins SIRT3 and SIRT5, and increased abundance of the mono-ADP-ribosyl polymerases PARP3 and PARP4 (Fig. S4*D*). NR supplementation rescued levels of multiple NAD^+^-related proteins, including SIRT3 (Fig. S4*D*). However, despite lower SIRT3 and SIRT5 protein abundances, mitochondrial lysine acetylation and malonylation were not significantly altered in PD-fed HNKO mice (Figs. S3*G* and S4*F*), indicating maintained activity in SIRT3- and SIRT5-regulated pathways.

### Respiratory capacity is not changed in primary hepatocytes from PD-fed HNKO mice

**Figure 9:**
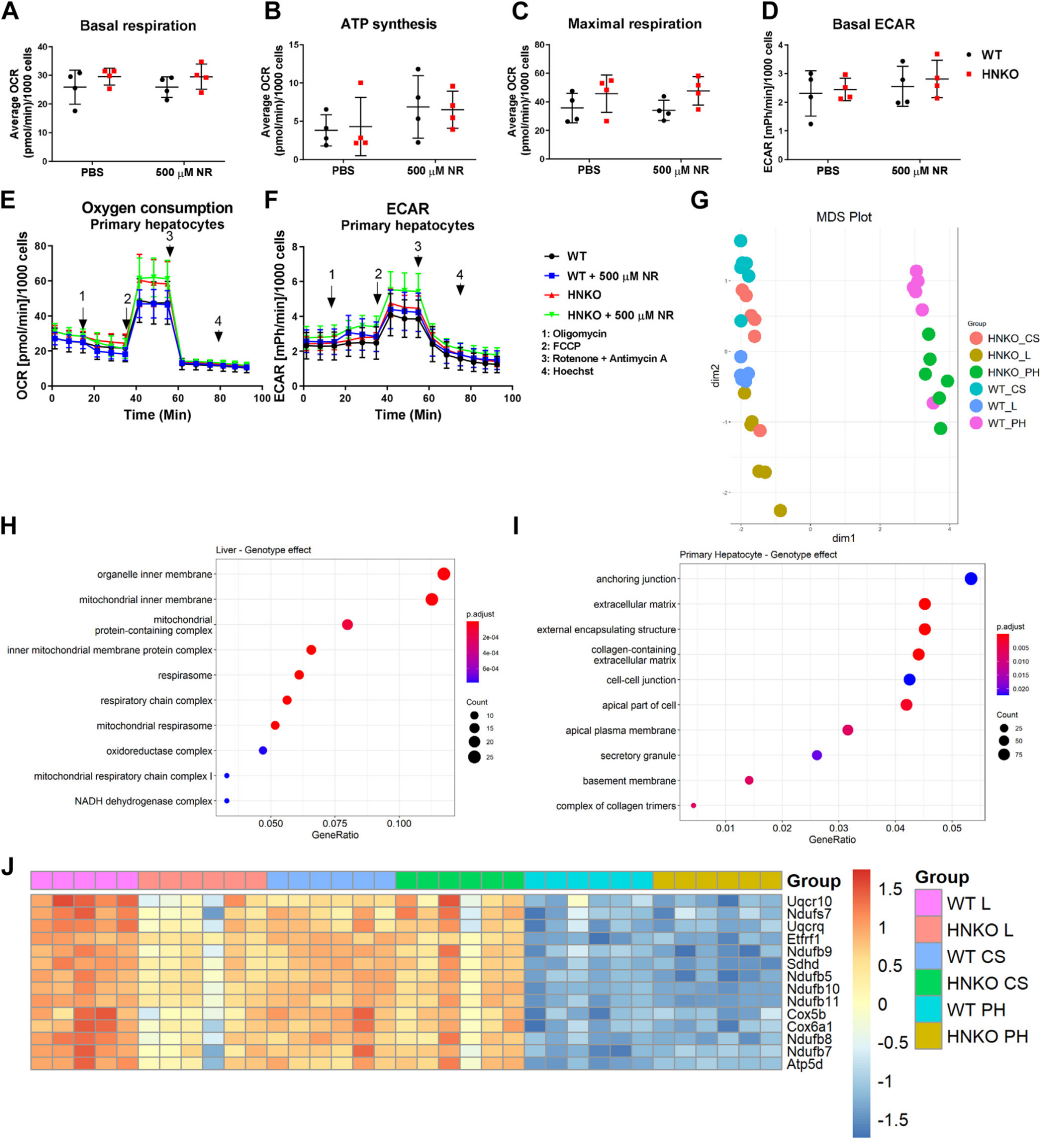
**Impaired maximal respiratory capacity in HNKO livers is not maintained in primary HNKO hepatocytes.** To further investigate the mitochondrial phenotype observed in HNKO mice, we isolated primary hepatocytes from HNKO mice and WT littermates fed PD for 5 to 7 weeks and measured oxygen consumption rate using the SeaHorse XFe96 extracellular flux analyzer. HNKO hepatocytes had no alterations in oxygen consumption rate, and RNA-Seq analysis of HNKO primary hepatocytes and full liver confirmed that isolation and culturing of primary hepatocytes negated the decreased expression of mitochondrial genes observed in HNKO livers. Average oxygen consumption rates (OCRs) for (*A*) basal respiration, (*B*) ATP synthesis, (*C*) maximal respiration, and (*D*) extracellular acidification rates (ECARs) for HNKO and WT primary hepatocytes cultured ±500 μM NR for 1 h before the assay. *E*, average OCR and (*F*) average ECAR from all runs. OCR and ECAR values were normalized to cell count, determined by Hoechst staining. n = 4. To investigate why mitochondrial function was retained in primary HNKO hepatocytes, we fed WT and HNKO mice PD for 3 to 5 weeks and compared transcription profiles from full liver (L), cell suspension before plating (CS), and primary hepatocytes cultured for 24 h (PH). *G*, MDS plot showing separation for dimensions 1 and 2, comparing gene expression profiles. Gene Ontology (GO) enrichment analysis for cellular component of genes with a differential expression between genotypes in (*H*) full liver and (*I*) primary hepatocytes. *J*, heatmap showing expression levels of genes involved in mitochondrial respiration with a significantly different expression between genotypes in full liver. n = 5 to 6. GO analysis was performed using the R package ClusterProfiler. MDS plots were generated using the R package Limma. Differential expression analysis was performed using the R package edgeR using a model of the form *expression ∼ Group.* HNKO, hepatocyte-specific *Nampt* knockout; MDS, multidimensional scaling; NR, nicotinamide riboside; PD, purified control diet.

To further elucidate the pathways underlying the impaired mitochondrial function observed in PD-fed HNKO mice, we isolated primary hepatocytes from PD-fed HNKO mice and WT littermates. First, we evaluated the respiratory capacity of the cells to assess whether impaired maximal respiratory capacity was maintained following hepatocyte isolation. As we previously observed that primary hepatocytes from chow-fed HNKO mice had no impairment of mitochondrial oxygen consumption (16), we cultured hepatocytes for 48 h before measurement to deplete NAD^+^ precursors in the media. We have previously observed that NAD^+^ levels decrease with culturing time in primary hepatocytes (16). To evaluate the acute effects of NAD^+^ repletion, we incubated half of the cells from each genotype with 500 μM of NR for 1 h before the respirometry measurements. We observed no change in oxygen consumption for basal respiration or for ATP synthesis, and contrary to our respirometry measurements from tissue biopsies, we observed no decrease in maximal respiratory capacity in HNKO hepatocytes (Fig. 9, *A*–*C* and *E*). Extracellular acidification rate (ECAR) was also unaltered between genotypes (Fig. 9, *D* and *F*), and NR supplementation caused no change in respiration or ECAR (Fig. 9, *A*–*F*). Hence, we detected no changes in respiratory capacity in primary hepatocytes isolated from HNKO mice and no effects of acute NR supplementation.

### Culturing of primary hepatocytes from HNKO mice causes major changes to the hepatocyte transcriptome

To investigate why the impaired respiratory capacity was not maintained in hepatocytes from PD-fed HNKO mice, we isolated hepatocytes from HNKO and WT mice following PD feeding and sampled a liver biopsy just as the liver perfusion was initiated. We also recovered a fraction of washed cells before plating and harvested the primary hepatocytes following 24 h culture. We isolated RNA from the six groups: WT and HNKO full liver, cell suspension, and primary hepatocytes and performed RNA-Seq. We observed a separation of primary hepatocytes compared with both cell suspension and liver, indicating that culturing caused major changes to the hepatocyte transcriptome (Fig. 9*G*). Surprisingly, a much larger number of genes were differentially expressed when comparing genotype effects in primary hepatocytes compared with liver (Fig. S5*D* and File S5). GO enrichment analysis on genes differentially expressed between HNKO and WT whole liver showed an enrichment of cellular component terms associated with mitochondrial function, whereas terms in primary hepatocytes were more associated with cell adhesion and extracellular matrix organization (Fig. 9, *H* and *I*). OXPHOS-associated proteins with a differential expression between genotypes were plotted as a heatmap, which revealed that expression was reduced in HNKO liver compared with WT liver (Fig. 9*J*). Interestingly, the decreased expression observed in HNKO liver seemed to be alleviated in HNKO cell suspension, indicating that the isolation process may cause an enrichment of healthy hepatocytes from the HNKO liver. OXPHOS expression was decreased in primary hepatocytes compared with liver and cell suspension, and no difference in expression between genotypes was observed. Hence, RNA-Seq data from this experiment demonstrate that the mitochondrial phenotype of HNKO livers is likely altered in cell culture, and that this model is not capable of recapitulating the phenotype observed *in vivo*.

### Single-nucleus sequencing reveals major transcriptional changes in hepatocytes from HNKO mice with necrosis

**Figure 10:**
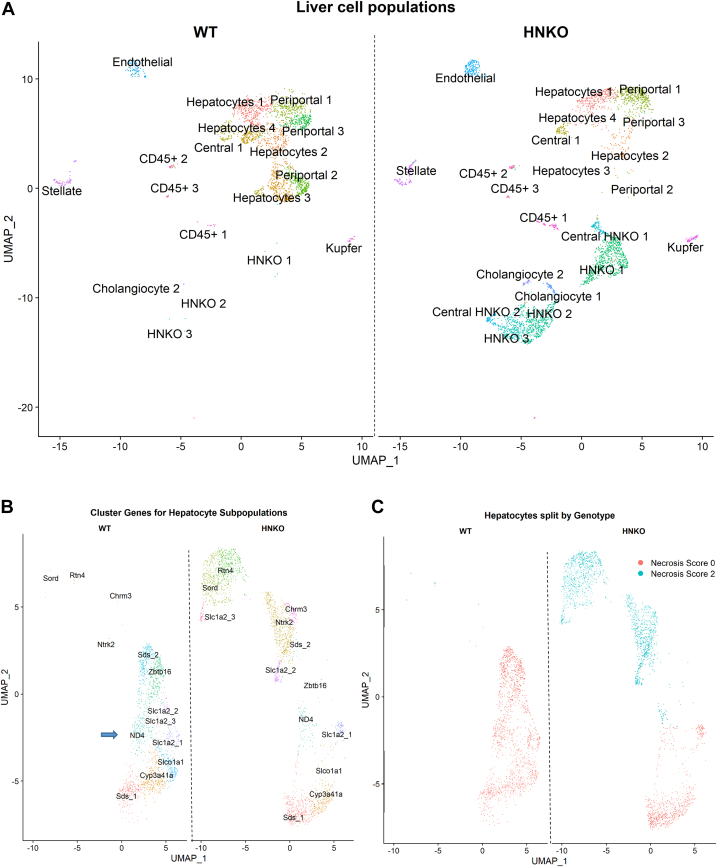
**Alterations in the HNKO transcriptome is associated with a positive histology score for necrosis.** Nuclei were isolated from three HNKO and three WT mice following 6 weeks of PD feeding, and RNA was sequenced from 4250 nuclei per sample. This analysis revealed that HNKO hepatocytes have a more heterogeneous transcription profile compared with WT hepatocytes and that heterogeneity amongst HNKO hepatocyte transcription profiles was found to be associated with histological necrosis scores. *A*, UMAP plot split by genotype, showing the clustering of cell types for WT and HNKO mice. Clusters were annotated to specific cell types based on the expression of cell-specific markers, identified from previous reports and the Human Protein Atlas. Hepatocytes were subsequently extracted and reclustered. *B*, UMAP projection showing clustering of hepatocytes, split by genotype. Clusters were annotated based on the nonpseudogene marker with the lowest FDR-adjusted *p*-value for that specific cluster. *C*, UMAP plot split by genotype, showing separation of clusters based on necrosis scores, obtained by scoring. n = 3. Cells were clustered, and markers were identified using the R package Seurat. FDR, false discovery rate; HNKO, hepatocyte-specific *Nampt* knockout; PD, purified control diet; UMAP, uniform manifold approximation and projection.

As the mitochondrial defect observed in HNKO liver could not be recapitulated in primary HNKO hepatocytes, we assessed alterations in the transcriptome of cells present in the livers of WT and HNKO mice to determine which cells were responsible for the transcriptomic signature of the whole liver. We isolated nuclei from three WT and three HNKO mice following 6 weeks of PD feeding (Fig. 6*A*) and subjected them to single-nucleus RNA-Seq. Cells tended to cluster strongly based on the donor mouse, and cells from two of the three HNKO mice separated clearly from the other cells (Fig. S6*A*). Clustering of expression profiles revealed the presence of 21 clusters, and cluster identities were determined by the presence of cell type–specific markers (Fig. S6*B*). Analyses confirmed that not only the majority of cells were hepatocytes but also identified endothelial cells, stellate cells, Kupffer cells, CD45+ cells, and cholangiocytes (Fig. 10*A*). Interestingly, cholangiocytes were primarily observed in HNKO livers, consistent with the presence of ductular reaction. Three hepatocyte clusters associated with HNKO hepatocytes clustered with cholangiocytes, suggesting a similar transcriptional profile, which could indicate transdifferentiation. Data from hepatocytes were extracted from the full dataset and reclustered. For the hepatocyte subanalysis, cells still largely separated based on donor mouse, regardless of genotype (Fig. S7*A*). Transcription profiles from HNKO hepatocytes appeared more heterogenous than for WT hepatocytes. Each cluster was assigned a gene identity based on the top marker genes identified through the FindMarkers function (Fig. 10*B* and File S6). Hepatocytes from WT and HNKO mice clustered according to known zonation markers such as *Sds* (periportal) and *Slc1a2* (central marker) (24). Inclusion of further metadata in the analysis revealed that HNKO hepatocytes in part separated based on their histological necrosis score (Figs. 10*C* and S9*B*). HNKO hepatocytes from livers with necrosis were characterized by expression of genes such as Reticulon-4 (*Rtn4*), sorbitol dehydrogenase (*Sord*), and BDNF/NT-3 growth factor receptor (*Ntrk2*). Among the hepatocytes without necrosis, we noted a smaller cluster associated with the gene *Nd4* (NADH-ubiquinone oxidoreductase chain 4), which is a subunit of the mitochondrial complex I (Fig. 10*B*, *blue arrow*). Cells from this cluster were mainly observed in WT livers. The top 20 marker genes for this cluster included COX1–3 and several subunits of complex I (Fig. S7*C*). Overall, we observed a lower average expression of these genes in hepatocytes from HNKO mice (Fig. S7*C*). Marker genes from this cluster were enriched for GO terms such as *electron transfer activity* indicating a subpopulation of oxidative hepatocytes (Fig. S7*D*). Together, these data suggest that the major transcriptional changes observed in HNKO livers occur in hepatocytes, and that these changes are not because of increased presence of myofibroblasts and immune cells.

## Discussion

Here, we show that hepatocyte-specific knockout of *Nampt* in the liver confers increased susceptibility to liver injury in mice, and that supplementation with the NAD^+^ precursors NR and NA can alleviate this phenotype and attenuate inflammation. We further demonstrate that HNKO livers from PD-fed mice have decreased respiratory capacity, and impaired abundance of mitochondrial proteins and oxidoreductases, which is normalized by NR. These results support the idea that NR can alleviate liver fibrosis, and they are in line with previous data showing NR attenuates high-fat, high-sucrose–induced, and CCL_4_-induced liver fibrosis in mice (2, 25). A main finding from this study is that the liver appears to have a threshold requirement for NAD^+^ and that fibrosis severity correlates with hepatic NAD^+^ content.

We demonstrate that loss of *Nampt* increases susceptibility toward damage from MCD diet, leading to an increase in plasma ALT activity and a decrease in lean mass. Sirius red and H&E stainings revealed that MCD-fed HNKO mice had severe liver damage, characterized by fibrosis, necrosis, ductular reaction, and periportal inflammation. To our surprise, a similar phenotype was observed in the PD-fed HNKO control group. We previously reported that HNKO mice had no noticeable phenotype, despite a decrease in liver NAD^+^ levels (16), and we had performed histological examinations of HNKO livers and found no obvious signs of liver injury. We therefore hypothesized that the phenotype was induced by PD due to a lower content of NAD^+^ precursors in that diet. However, fibrosis was present after 3 days of PD feeding, suggesting either rapid induction or that fibrosis was present before PD feeding. HNKO mice had increased abundance of plasma biomarkers previously associated with liver damage at all investigated time points, confirming the presence of ongoing liver damage (20). We subsequently found that fibrosis was also present in some HNKO mice when they were fed chow diets with a higher content of NAD^+^ precursors, but we also noted a negative correlation between liver NAD^+^ content and fibrosis severity, confirming that fibrosis is associated with low NAD^+^ levels in HNKO mice. Overall, our data suggest that liver injury occurs in HNKO mice when liver NAD^+^ levels are sufficiently decreased.

Grain-based diets show batch variability due to differences in crop quality (26). To avoid this confounding factor, we continued our experiments with the PD. We show that a relatively minor increase in dietary NA content in the PD (from 29 to 75 mg/kg) can prevent portal inflammation, necrosis, and ductular reaction in PD-fed HNKO mice. Assuming that a 25 g mouse eats 4.4 g of feed daily (27), this is an average consumption of 13.2 μg NA/g BW/day for PD+NA, *versus* 5.1 μg NA/g BW/day for PD. Hence, the dietary NA intake was minor compared with the commonly applied NR dose of 400 μg/g BW/day (2, 8). Our findings that inflammation can be attenuated and fibrosis partly reversed by NR show that NR can protect against liver damage. However, our data also suggest that high doses of NR were associated with a decrease in hepatic ATP content in NR-fed mice irrespective of genotype, indicating that the high amount of NR could be challenging for the hepatocytes. A recent report showed that NR supplementation caused an inhibition of sirtuin activity in cardiac tissue due to an accumulation of nicotinamide, though this was not observed in liver (28). Another report found that a lower dose of NR was more effective at improving the metabolic phenotypes of high-fat diet-fed mice, indicating that beneficial effects of NR might be achieved at lower doses (29).

Liver injury in HNKO mice is present only in the portal area. Additional work is required to determine how closely the HNKO phenotype resembles other models of periportal fibrosis development. We initially noticed that the fibrosis pattern resembled the initial stages of bile duct ligation (22), but we found no evidence of impaired bile flow. We also noted that the pattern also bears resemblance to fibrosis induced by CCL_4_ treatment (30). PD-fed HNKO mice could be a comparable and more convenient alternative to CCL_4_ treatment, with less stress inflicted upon the animal. A recent study found that liver-specific *Nrk1* knockout mice are susceptible to fibrosis development when fed HFD (31). However, in that model, fibrosis was not periportal, and NAD^+^ content was only slightly reduced in the knockout group, suggesting a distinct phenotype compared with the HNKO mice. We observed an increased expression of several cholangiocyte and oval cell markers in HNKO livers. It has previously been suggested that these cell types can contribute to liver regeneration when hepatocyte proliferative capacity is impaired (21). Hence, bile duct proliferation and periportal fibrosis could be a compensatory and regenerative response to alleviate the *Nampt*-deficient hepatocytes. This was further supported by our single- nucleus RNA-Seq data. Several *Acaa1b*^*+*^*–Scd1*^*+*^*–Rbp4*^+^ hepatocytes from HNKO mice clustered with *Ctnnd2*^*+*^ and *Spp1*^*+*^ cholangiocytes, suggesting a similar transcriptional profile. Another possibility is that the colocalization of fibrosis with the bile ducts could be a consequence of a differential reliance of NAMPT-mediated NAD^+^ salvage across the liver lobule. A previous study observed that several enzymes from the *de novo* pathway appear to be zonated toward the central vein, supporting this hypothesis (32). Future studies will focus on elucidating the mechanism for this zonation in the injury pattern of the HNKO mice.

To elucidate which pathways are responsible for the observed phenotype in HNKO livers, we mapped the HNKO transcriptome and proteome and found major changes in both. However, the presence of fibrosis and the large number of infiltrating cells meant that when differentially expressed genes and differentially abundant proteins were subjected to enrichment analyses, most significant terms were associated with extracellular matrix organization and cytoskeletal rearrangements. Further analysis revealed that livers from HNKO mice had a significant decrease in maximal respiratory capacity and decreased abundance of mitochondrial proteins across multiple cohorts and experiments. Respiratory capacity was found to correlate to SDH activity, which was decreased in HNKO mice. Interestingly, the mitochondrial defects were not associated with an increased production of ROS or an altered content of liver mitochondria. When we looked further at differentially abundant proteins involved in oxidation–reduction processes, we noted that a large number of these proteins use NAD(H) or NADP(H) as cofactors. We observed a decreased abundance of multiple dehydrogenases in HNKO livers, including aldehyde dehydrogenases, steroid dehydrogenases, isocitrate dehydrogenases, and hydroxyacyl-coenzyme A dehydrogenases. Hence, the proteomics data suggest an impaired capacity in HNKO mice for dehydrogenase reactions, potentially caused by decreased cofactor availability. A future research direction will be to determine the effects of reduced NAD^+^/NAMPT for detoxification or other electron transfer–requiring reactions.

The proteomics analysis revealed that HNKO livers had altered abundance of several proteins involved in NAD^+^ biosynthesis, including NMNAT3 and multiple proteins from the *de novo* NAD^+^ synthesis pathway. We were surprised to see a decreased abundance of the latter, as we expected HNKO mice to sustain liver NAD^+^ content largely through the *de novo* pathway. We observed increased abundance of the mono-ADP ribosyltransferases PARP3 and PARP4 and decreased abundance of NADK and the mitochondrial sirtuins SIRT3 and SIRT5. We hypothesized that the mitochondrial sirtuins could be causing the decreased abundance of NAD^+^ consumers. SIRT3 has a relatively high K_m_ value of 880 μM, which makes it susceptible to decreases in intracellular NAD^+^ content (33). Furthermore, SIRT3 has been shown to regulate transcriptional levels of antioxidants such as SOD2 and glutathione peroxidase 1 (34), and both those genes had decreased expression in HNKO liver. SIRT3 has also been shown to cause nuclear translocation of the transcription factor FOXO3a (35), demonstrating that SIRT3 can regulate gene transcription. SIRT5 has a similar high K_m_ value of 980 μM and could therefore also be sensitive to decreased NAD^+^ levels in HNKO mice (36). However, we observed no alterations in mitochondrial protein acetylation or malonylation levels, suggesting normal SIRT3 and SIRT5 activities in HNKO livers. Though the decreased abundance of several NAD^+^-dependent proteins could indicate a coordinated response, we found no conclusive evidence to support this idea.

Interestingly, the impaired maximal respiratory capacity observed in HNKO livers was not present in primary HNKO hepatocytes, and mitochondrial function was not affected by NR supplementation for either genotype. We further demonstrated that the transcriptional signature observed in full liver was altered during the major transcriptional changes induced by hepatocyte plating and culturing. Expression of OXPHOS subunits was not significantly altered between WT and HNKO primary hepatocytes, which may explain why maximal respiratory capacity was maintained in HNKO hepatocytes. A recent report investigating the effects of NR for liver regeneration found improved mitochondrial oxygen consumption when NR was administrated during culture in glucose-free Dulbecco's modified Eagle's medium (DMEM) before respirometry analysis (37). Hence, culturing conditions of primary hepatocytes may not have been severe enough to display marked effects on mitochondrial function in our hands. Differentially expressed genes in full HNKO liver were associated with mitochondrial organization and the respirasome, whereas HNKO hepatocytes had a differential expression of genes involved in extracellular matrix organization and cell junction maintenance. This suggests that NAD^+^ deficiency forces the hepatocytes to adapt differently to culture conditions.

Few proteins had altered abundance in WT mice following NR, supporting previous observations that in the absence of a metabolic challenge, WT mice have little benefit from NR (8). NR had more pronounced effects in the HNKO group, where NR restored the abundance of proteins associated with oxidoreductase reactions. The restoration of *oxidoreductase activity* proteins was accompanied by decreased inflammation scores, suggesting that damage is alleviated, preventing further inflammation and extracellular matrix deposition. Single-nucleus sequencing revealed that major transcriptional changes were associated with the presence of necrosis in HNKO mice and supported decreased expression of OXPHOS genes in HNKO hepatocytes. Hence, these data further suggest that the transcriptional changes in HNKO livers occur in hepatocytes and that they are not because of infiltration of other cell types, like immune cells and myofibroblasts.

**Figure 11:**
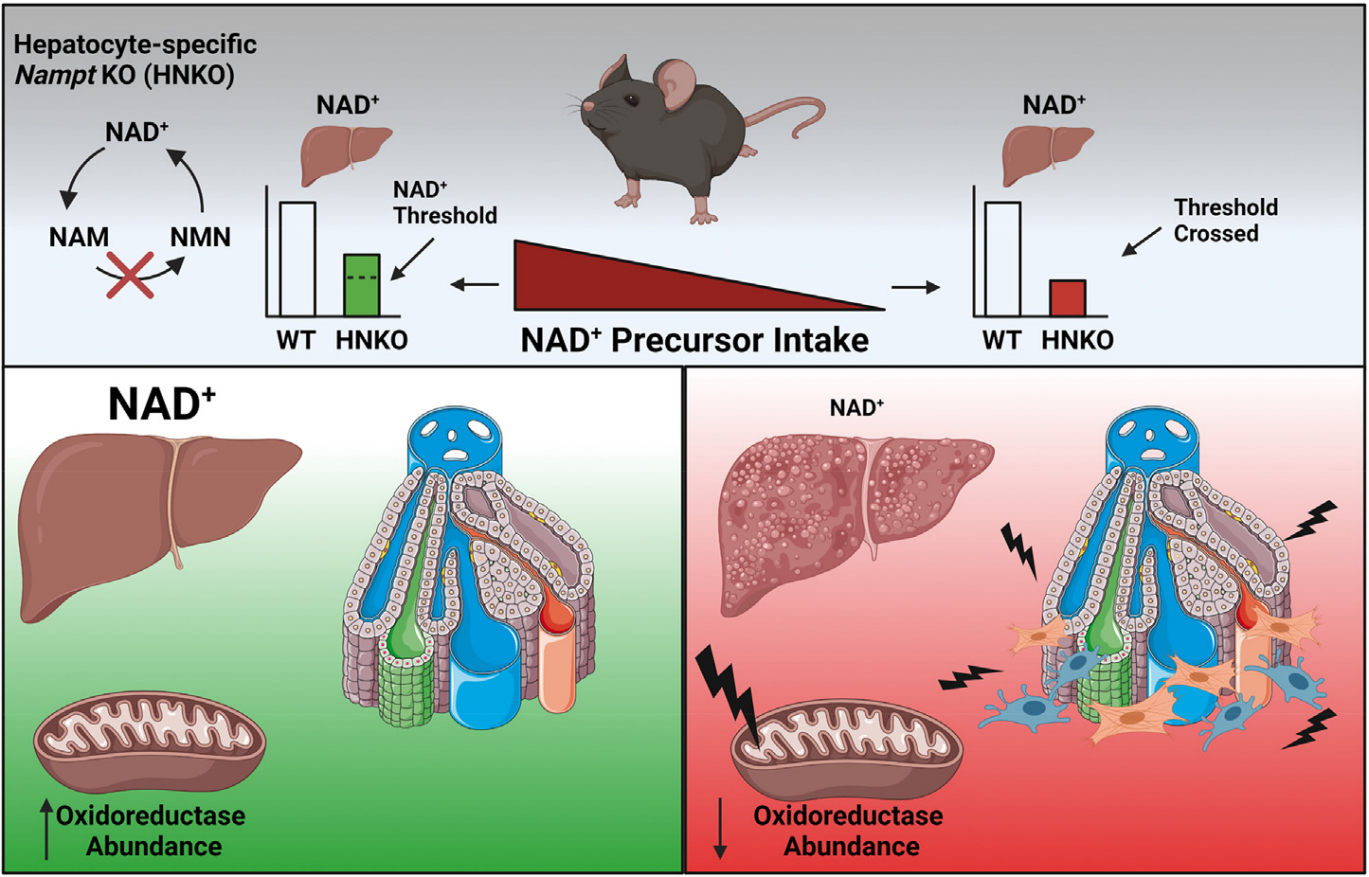
**Liver injury in HNKO mice is determined by hepatic NAD**^**+**^**content and associated with decreased oxidoreductase abundance.** Graphical summary and interpretation of key findings. NAD^+^ precursor intake protects HNKO mice from developing liver injuries, suggesting the presence of an NAD^+^ threshold required to maintain a healthy liver function. If this threshold is crossed, HNKO mice develop periportal inflammation and stellate cell activation. The phenotype is associated with decreased abundance of mitochondrial oxidoreductases. HNKO, hepatocyte-specific *Nampt* knockout.

Overall, our results demonstrate that loss of NAD^+^ salvage confers increased susceptibility to liver injury, and that this injury can be prevented and attenuated by NAD^+^ precursor supplementation, which is associated with an increased abundance of mitochondrial proteins involved in oxidoreductase reactions. Our data suggest the presence of an NAD^+^ threshold in the liver for fibrosis susceptibility, proving a link between liver fibrosis and hepatic NAD^+^ content (Fig. 11).

## Experimental procedures

### Chemicals and reagents

Unless otherwise noted, all chemicals and reagents were purchased from Merck.

#### Mouse experiments

##### Mouse strain and housing

HNKO mice (*Nampt*^fl/fl^ [*Nampt*^TM1Jtree^ (38)] and *Alb*-Cre^+/−^) and floxed WT littermates were generated and housed as previously described (16). From weaning, mice were fed a fortified breeding chow *ad libitum* (1319; Altromin).

##### MCD feeding experiment

Female HNKO and WT mice (10–16 weeks of age) were randomized into four groups based on lean mass. Mice were either fed an L-amino-acid defined, low MCD (A06071302; Research Diets) (39) or a matched, purified, L-amino acid defined low-fat PD (A06071314; Research Diets) for 3 weeks. Body composition was determined before and after the diet intervention using an NMR scanner (EchoMRI 4-1; EchoMRI). Before sacrifice, mice were fasted for 4 h from ZT = 3. Mice were sedated with pentobarbital as previously described (16) and killed by cardiac puncture with a heparin-coated needle. Blood was transferred to tubes with EDTA and was centrifuged at 6000*g* at 4 °C for 6 min. Plasma was transferred to a new tube and stored at −80 °C. Part of the hepatic median lobe was fixed in 4% paraformaldehyde (PFA) for histology. The remaining liver was snap frozen and stored at −80 °C. The frozen liver was crushed in liquid nitrogen before further analysis.

##### PD time course feeding experiment

Female HNKO and WT mice (9 weeks of age) were randomized into four groups and fed PD for 3, 6, 12, or 21 days. Mice were sedated with pentobarbital, and plasma was prepared as described previously. Part of the median lobe was fixed in 4% PFA, and the remaining liver was snap frozen in liquid nitrogen and stored at −80 °C before further analysis.

##### Chow *versus* PD feeding experiment

Female HNKO and WT mice (8–13 weeks of age) were randomized into four groups and fed either PD, FBD/chow (1319F; Altromin), SBD (1319; Altromin) or SMD (1329; Altromin) for 21 days. Mice were sedated with pentobarbital, and plasma was prepared as described previously. Part of the median lobe was fixed in 4% PFA, and the remaining liver was snap frozen in liquid nitrogen and stored at −80 °C before further analysis.

##### NR prevention and NA supplementation experiments

For the 3-week NR experiment, female HNKO and WT littermates, 8 to 11 weeks of age, were fed PD for 3 weeks. NR was supplied in drinking water (16). NR was dosed as 1.9 g/l, corresponding to a dose of 400 mg/kg BW/day for a 20 g mouse, based on an average daily water intake of 4.4 ml/mouse (16). Fresh water ± NR was supplied every third day. For the NA prevention study, female HNKO mice and WT littermates at 8 to 12 weeks of age were fed either normal PD (containing 29 mg NA/kg diet) or NA-enriched PD containing 75 mg NA/kg diet (A18031901; Research Diets) for 3 weeks. Mice were sacrificed, and tissues were prepared as described for the MCD feeding experiment.

##### NR intervention and bile duct catheter insertion

For the 6-week NR intervention experiment, female HNKO mice and WT littermates (8–12 weeks of age) were fed PD for 6 weeks. After 3 weeks, mice were split into two groups, receiving either normal drinking water or NR water as described previously. On the day of sacrifice, mice were anesthetized with isoflurane (2.5% for induction and 1.5% for maintenance) and placed in dorsal recumbency on a 37 °C heat pad for thermal support. Following a midline laparotomy, the gall bladder and bile ducts were exposed by atraumatic retraction and fixation of the xiphoid process and the left and right lateral liver lobes. Throughout the surgery, the abdominal cavity was kept moist with plenty of warm sterile saline. The distal common bile duct (CBD) was isolated using blunt dissection, and two 7-0 silk ligatures were placed around it. The CBD was occluded by tightening the distal ligature, whereas the proximal ligature was left loosely in place. With Iris scissors, an incision was made in the CBD on the proximal side of the occluding ligature, and a 0.75 French polyurethene catheter (10 cm piece of MRE010 tubing; Braintree Scientific) was inserted and advanced approximately 2 mm. The proximal ligature was tightened around the catheter and CBD wall to prevent leakage, and the placement of the catheter was adjusted to ensure that the tip was in the CBD and did not block the flow from the hepatic ducts. Once in place, the distal ligature was tied around the catheter to secure it, and an occluding ligature was tied around the cystic duct to block the flow of bile into the gall bladder. The free end of the catheter was placed in a preweighed collection tube, which was kept on ice next to the mouse. To minimize the effects of gravity on the bile flow, the top of the collection tube was positioned in the plane of the mouse, ensuring that the catheter was kept in a horizontal direction. Finally, the abdominal incision was closed with staples to prevent dehydration and hypothermia during the collection period. As soon as the bile reached the collection tube, a timer was started and the collection was continued until at least 10 μl had been obtained, which was achieved within 15 to 30 min for all mice. Throughout the collection period, the flow rate was monitored by observing the rate of droplet formation at the catheter tip, which did not change over time for any of the mice. Once the collection was complete, the tubes were weighed again and the samples were stored at −80 °C. The bile secretion rate was calculated as mg bile/h (mg/g/h) by subtracting the weight of the tube from the total sample weight and normalizing for the collection time. Following collection, mice were killed by cardiac puncture. The left lobe was fixed for histology, and the remaining liver was snap frozen in liquid nitrogen.

#### Histology and scoring

Formaldehyde-fixed livers were dehydrated, embedded in paraffin, and cut into 4 μm sections, which were mounted on glass slides. For H&E staining, sections were stained with hematoxylin and eosin. For Sirius red (40), sections were incubated overnight in Bouin's fixative and incubated with Weigert's hematoxylin followed by aqueous picric acid with DirectRed80. For immunohistochemistry, sections were dewaxed and pretreated by 10 min of microwave boiling in EGTA buffer (pH = 9), followed by blocking in 2% bovine serum albumin (BSA). Sections were incubated overnight at 4 °C with primary antibodies in relevant dilutions. Antibodies against the following targets were used: CD45 (1:1600, ab10558; Abcam), CK-19 (1:400, ab52625; Abcam), Ki67 (1:2000, ab16667; Abcam), SMA (1:2000, ab5694; Abcam), and COXIV (1:3200, ab16056; Abcam). On day 2, sections were incubated with biotin-antimouse or anti-rabbit antibody, followed by avidin–biotin–complex (Vectastain; Vector Laboratories) and diaminobenzidene and counterstained with hematoxylin. Images were acquired using either a Cool Snap camera (Teledyne Photometrics) or a Zeiss Axio Scan Z.1 Slide Scanner (Zeiss). Stained areas were quantified using Image Pro 7 software (Media Cybernetics) for CK-19, CD45, and SMA, and Zeiss Zen Intellesis software (Zeiss) for COXIV and Sirius red stainings. For scoring, slides were blinded and scored by two of the coauthors. Each slide was given a score as an average of the two individual assessments, allowing for “half” scores. Slides were scored according to the following parameters: Fibrosis: 0, none; 1, perisinusoidal or periportal; 2, perisinusoidal and periportal; 3, bridging fibrosis; 4, cirrhosis. Portal inflammation: 0, none or minimal and 1, the presence of portal inflammation. Hepatocyte necrosis: 0, none; 1, few cells; and 2, prominent/many cells. Ductular reaction: 0, none; 1, some observed; and 2, severe. All displayed images were acquired at 10× magnification.

#### Biochemical analysis

All absorbance, fluorescence, and luminescence-based assays were quantified using a Hidex Sense plate reader (Hidex). Hepatic triglyceride content was quantified using a kit (KA0847; Abnova) with modifications as previously described (16). Plasma ALT levels were determined by a commercial kit (ab105134; Abcam). NAD^+^, NADP^+^, and NADPH levels in liver tissue were determined using an enzymatic cycling assay as previously described (41). Citrate synthase activity was determined as previously described (16). Protein carbonylation levels were determined in liver lysates (prepared as described for Western blot analyses later) using a commercial kit according to the manufacturer's instructions (MAK094; Sigma-Aldrich). ATP levels were determined from app. Twenty milligrams of liver tissue homogenized in 400 μl 0.6 M perchloric acid, using a commercial kit according to the manufacturer's instructions (A22066; ThermoFisher Scientific).

#### SDH activity

SDH activity was determined as described previously (42). Liver tissue (20–30 mg) was homogenized in 500 μl buffer (50 mM Tris–HCl, 1 mM EDTA, 0.1% Triton X-100, and pH 7.4) before being treated to three rapid freeze–thaw cycles. Samples were spun down for 10 min at 5000*g*, 4 °C, and 1:6 dilution of the supernatant was used in the activity assay. About 20 μl of the diluted sample was mixed with 230 μl of the reaction buffer (50 mM KH_2_PO_4_, 20 mM succinate, 2.5 mM sodium cyanide, 2.5 μM antimycin A, 0.45 mM phenazine methosulphate, and pH 7.4) in a 96-well plate and incubated for 5 min at 30 °C. Reaction was initiated by addition of 50 μl of 0.72 mM dichlorophenolindophenol, and absorbance was followed at 600 nm for 10 min using a Hidex Sense plate reader (Hidex). Enzyme activity was normalized to protein content determined using the Bicinchoninic Acid Assay (23227; ThermoFisher Scientific).

#### Primary mouse hepatocyte experiments

##### Extracellular flux analysis

Primary mouse hepatocytes were isolated from male HNKO and WT mice fed a PD for 5 to 7 weeks, beginning from 12 to 13 weeks of age. Primary hepatocytes were isolated as previously described (16) and seeded as 12,000 living cells/plate in collagen-coated XF96-seahorse plates from the SeaHorse XFe96 Fluxpak (102416-100; Agilent). After isolation and attachment, cells were incubated for 48 h at 37 °C with 5% CO_2_ in culture medium (MEM-199; catalog no.: 41150 [Thermo Fisher Scientific] with 0.5% fetal bovine serum, 1% penicillin/streptomycin, 1 μM dexamethasone, and 1 nM insulin). On the day before the extracellular flux analysis, 20 ml SeaHorse calibrant solution (100840-000; Agilent) and a sensor cartridge immersed in 200 μl sterile water/well were placed in a non-CO_2_ incubator at 37 °C and incubated overnight. One hour before measurement, cells were removed from the incubator, and culture medium was replaced with XF DMEM (103575-100; Agilent) supplied with 2 mM glutamine, 1 mM pyruvate, and 10 mM glucose ±500 μM NR. Cells were incubated for 1 h at 37 °C without CO_2_. Water was discarded from the sensor cartridge and replaced with the Calibrant Solution. Before measurement, a bright-field image was acquired for each well using a Cytation 1 Cell Imaging Multimode Reader (BioTek) to visually inspect cells. These images were subsequently used to verify low cell count in aberrant wells with low OCR. Injection ports on the cartridge were loaded with compounds as previously described (16) for the following final concentrations in wells:1 μM oligomycin (O4876; Sigma), 0.4 μM FCCP (C2920; Sigma), 2 μM rotenone + antimycin A (R8875 + A8674; Sigma). Compounds were added sequentially, followed by three cycles of measurements. Finally, injection port D was loaded with XF DMEM containing 0.1% Hoechst 33342 (generously donated by the Flow Cytometry & Single Cell Core Facility at the University of Copenhagen). Hoechst solution was injected after measurements, and cells were incubated with Hoechst for 30 min. Stained nuclei were counted using the Cytation 1 Cell Imaging Multimode Reader, and OCRs + ECARs were normalized to cell number for individual wells. Oxygen consumption for ATP synthesis and maximal respiration was calculated as previously described (16).

##### Preparation of hepatocytes for liver *versus* primary hepatocyte experiment

Female HNKO mice and WT littermates were fed PD for 3 to 5 weeks from 10 to 12 weeks of age and were used for primary hepatocyte isolation. Mice were sedated with avertin, and primary hepatocytes were isolated as previously described (16). When blood had been cleared from the liver following infusion with Hank's balanced salt solution with EGTA, a suture was placed and tightened around the median lobe, and a piece of the median lobe was cutoff and snap frozen in liquid nitrogen. After collagenase digestion, the median lobe was removed, and the remaining liver was dissociated in plating media and passed through a 100 μm strainer to release hepatocytes into suspension. Cells were washed and filtered as previously described (16). About 2 ml of the cell suspension was transferred to a 2 ml tube and centrifuged at 100*g* for 2 min. The supernatant was removed, and cell pellet was snap frozen in liquid nitrogen. One million living cells/well were plated in a collagen-coated 6-well plate for each genotype. Plated cells were allowed to attach for a minimum of 2 h before media were removed and switched to culture media, as previously described. After 24 h of culturing, media were removed, and cells were washed in PBS before 200 μl of trypsin–EDTA was added. Cells were incubated at 37 °C for a maximum of 5 min and trypsin neutralized in 800 μl culture media. Cells were transferred to a 1.5 ml tube and centrifuged at 200*g* for 5 min. Media were aspirated, and pellets were snap frozen. Liver samples (L), cell suspension samples (CS), and cultured primary hepatocyte samples (PH) were stored at −80 °C before RNA isolation for RNA-Seq.

#### RNA-Seq

##### Library preparation for RNASeq

Total RNA was isolated using Trizol (Thermo Fisher Scientific) followed by purification with an RNeasy mini kit (Qiagen) according to the manufacturer's protocol. Total RNA-Seq libraries were prepared using the Illumina TruSeq Stranded total RNA Gold protocol (Illumina). The total RNA (500 ng) was depleted of rRNA by RiboZero beads, fragmented, and cDNA was synthesized using SuperScript III Reverse Transcriptase (Thermo Fisher Scientific). cDNA was adenylated to prime for adapter ligation, and after a cleanup using AMPure beads (Beckman Coulter), DNA fragments were amplified using PCR followed by a final cleanup. Libraries were quality controlled using a Bioanalyzer instrument (Agilent Technologies).

##### Alignment and data analysis

Libraries were subjected to 51-bp paired-end sequencing on a NovaSeq 6000 system (Illumina). A total of 1.1 billion reads were generated for the time course experiment and 883 million reads for the liver *versus* primary hepatocytes experiment. STAR, version 2.7.2b (for the time course experiment) and version 2.5.2b (for the liver *versus* primary hepatocytes experiment (43)) was used to align sequence reads onto the GRCm38 mouse genome assembly, with the GENCODE version M22 gene model (44), retrieved on June 27, 2019 (for the time course experiment) and July 31, 2018 (for the liver *versus* primary hepatocytes experiment). After mapping, reads were summarized onto genes by featureCounts, version 1.6.4 (45) with the same gene model as when mapping. Fragments were only counted where both ends of the fragment were aligned. Two samples in the time course experiment and one sample in the liver *versus* primary hepatocytes experiment had skewed GC (nucleotides guanine and cytosine) distributions, high levels of sequence duplication, lower rates of alignment, and were found to cluster by themselves in multidimensional scaling plots and with hierarchical clustering. These samples were excluded from further analysis. The quasi-likelihood tests found in the R package edgeR (46) (version 3.26.0 for time course and version 3.32.1 for liver *versus* primary hepatocytes) were used for differential expression testing. For both RNA-Seq experiments, the model *expression* ∼*Group* was used. Genes with a differential expression were further analyzed using GO enrichment analysis through the R package ClusterProfiler (47). Dotplots with GO terms display the 10 terms with the highest gene ratio or all GO terms when fewer than 10 significant terms were detected.

#### Single-nucleus RNA-Seq

##### Nucleus isolation

Single-nucleus RNA-Seq was performed by the Single-Cell Omics platform at the Novo Nordisk Foundation Center for Basic Metabolic Research at the University of Copenhagen. A suspension of single nuclei was prepared from three WT and three HNKO samples from the NR intervention cohort. Tissue was crushed on liquid nitrogen, and a small amount (∼2–3 mm^3^) was transferred to a nonstick Eppendorf tube, and 1.5 ml of lysis buffer (pure lysis buffer [L9286-180 ml; Sigma], about 0.1% DTT (43816-10 ml; Sigma), and 1% Triton (T1565-1.7 ml; Sigma) were added. Samples were incubated on ice for 5 min and carefully pipetted up and down during the incubation. Samples were centrifuged for 5 min at 750*g* at 4 °C, and the supernatant was removed. Pellet was resuspended in 1 ml nuclei buffer (PBS; w/o MgCl₂ and CaCl₂ [20012068; Invitrogen]), 1% BSA (SRE0036-25 ml; Sigma), 2 mM MgCl_2_ (M1028-100 ml; Sigma), and 0.1 % Protector RNase inhibitor 40 U/μl (3335399001; Sigma), and the solution was passed through a 40 μm strainer (43-10040-40; pluriSelect). Samples were incubated for 10 min on ice and centrifuged for 5 min at 750*g* at 4 °C. The supernatant was removed, and the pellet was resuspended in 110 μl nuclei buffer. About 10 μl was recovered and mixed with 10 μl trypan blue solution (93595-50 ml; Sigma) for counting nuclei in a counting chamber. For each sample, the remaining 100 μl were added 1 μl of individual TotalSeq-A antibodies (BioLegend) from 0.5 μg/μl stocks to enable multiplexing (48). Samples were incubated for 30 min on ice, and each sample was added to 1 ml nuclei buffer. Samples were centrifuged 10 min at 1000*g* at 4 °C, and the supernatants were discarded. Pellets were resuspended in 200 μl Nuclei Freeze Buffer (EZ Nuclei Storage Buffer [NUC101; Sigma], 0.1% BSA [SRE0036-25 ml; Sigma] and 2 mM MgCl_2_ [M1028-100 ml; Sigma]) and stored at −80 °C until sorting.

##### Sequencing

Using a SH800S Cell Sorter (SONY), 4250 nuclei from each of the six samples was sorted and pooled for generation of libraries for small nuclear RNA (snRNA)-Seq using the 10× Genomics Chromium single-cell 3′ reagent kits (version 3.1). Libraries were sequenced on an Illumina NovaSeq 6000. Fastq files were generated using cellranger 5.0.1. Reads were mapped with Alevin (49) onto the GRCm38 mouse genome assembly, with the GENCODE, version M25 gene model and imported as a Seurat object from the Alevin Output file. The RNA-Seq datasets were uploaded to the Gene Expression Omnibus database. Data were further processed by Seurat, version 4.0 (50). Raw data were filtered to retain nHTOcounts with more than 100 and less than 1250 counts. Counts were HTO normalized and demultiplexed, and negatives were removed. Singles were subset, and data were further subset by RNA features, where singles with more than 0 and less than 5500 features were retained. Data were normalized using the SCTransform function. Data were subject to principal component analysis dimensionality reduction for 35 principal components, followed by uniform manifold approximation and projection dimensional reduction technique for 1:35 principal components. Nearest neighbors were calculated, data were clustered, and marker genes were identified for each cluster through the FindMarkers function. Clusters were linked to specific cell types based on markers identified in previous publications (24, 51, 52) and by identifying cell-specific cluster markers through the Human Protein Atlas (53). For hepatocyte-specific analysis, hepatocyte clusters were subset and subject to principal component analysis + uniform manifold approximation and projection reduction, neighbor calculation, and clustering. Positive markers were identified for each cluster, and clusters were assigned identities based on nonpseudogene marker genes with highest false discovery rate (FDR)-adjusted *p*-value. Marker genes from the ND4 cluster were analyzed using ClusterProfiler as described above.

#### Proteomics

The LC–MS instrumentation for proteomics analysis consisted of an EASY-nLC 1200 system coupled to a nano-electrospray ion source and a Q Exactive HF-X Orbitrap (all Thermo Fisher Scientific). Purified peptides were separated on 40-cm HPLC columns (ID: 75 μm; in-house packed with ReproSil-Pur C18-AQ 1.9 μm resin [Dr Maisch GmbH]). Column temperature was kept at 60 °C using an integrated column oven (PRSO-V2; Sonation). Detailed LC–MS/MS analysis is described later. All samples were acquired in single-shot proteomics with one technical replicate.

##### Plasma proteomics

Proteome analysis of plasma samples was performed on an automated liquid handling system (Agilent Bravo) in a 96-well plate format as previously described (20, 54). Proteins were denatured, reduced, alkylated, digested, and peptides were purified on StageTips (55) using reagents from the PreOmics “iST” Kit (P.O. 00001; PreOmics GmbH). Purified peptides were analyzed by LC–MS/MS in data-independent acquisition (DIA) mode. Dried peptides were dissolved and sonicated in 5% acetonitrile/0.1% TFA, and 500 ng of purified peptides were injected for LC–MS/MS analysis. Peptides were loaded in buffer A (0.1% formic acid) and eluted with a 38-min linear gradient from 3 to 35% of buffer B (0.1% formic acid, 80% [v/v] acetonitrile), followed by a 5-min increase of buffer B to 98%, and a 2-min wash at 98% of buffer B at a flow rate of 450 nl/min. The DIA–MS method consisted of an MS1 scan from 350 to 1650 m/z range at a resolution of 120,000 followed by 22 DIA segments at a resolution of 30,000 in MS2 scans (File S7). The automatic gain control (AGC) target is 3 × 10^6^ for both MS1 and MS2 scans, with a maximum injection time of 50 and 54 ms for MS1 and MS2 scans, respectively. Fragmentation of precursor ions was performed by higher–energy collisional dissociation with a normalized collision energy of 27%.

##### Liver proteomics

Snap-frozen liver tissue samples were homogenized in 150 μl of SDC reduction and alkylation buffer (PreOmics GmbH) using a polytron homogenizer. The homogenate was then heated at 95 °C for 10 min, 1200 rpm on a thermos mixer (Eppendorf) to denature proteins, subsequently sonicated using a water bath sonicator (Diagenode Bioruptor) at full power for 30 cycles with 30 s intervals. Protein content was determined by tryptophan assay, and a volume containing 50 μg of protein was digested overnight with trypsin and LysC (1:50, μg of enzyme: μg of protein) at 37 °C, 1200 rpm on a thermos mixer. Digestion mixture was acidified to a final concentration of 0.1% TFA to quench the digestion reaction. Peptide concentration was estimated using Nanodrop (Thermo Fisher Scientific) and 20 μg of peptide mixture was purified by solid-phase extraction in a Stage-Tip format (SDB-RPS material, two 14-gauge plugs), washed with isopropanol/1% TFA and 0.2% TFA (200 μl each, centrifuged at 1500*g* with 3D-printed centrifuge block). Peptides were eluted with 60 μl of 80% acetonitrile/1% ammonia and dried at 60 °C using a SpeedVac centrifuge (Eppendorf; Concentrator plus). Dried peptides were dissolved and sonicated in 5% acetonitrile/0.1% TFA. Following preparation, peptide concentration was measured using Nanodrop, and 500 ng of purified peptides were injected for LC–MS/MS analysis. Peptides were loaded in buffer A (0.1% formic acid) and eluted with a linear 82 min gradient of 3 to 23% of buffer B (0.1% formic acid, 80% [mv/v] acetonitrile), followed by a 8 min increase to 40% of buffer B. The gradients then increased to 98% of buffer B within 6 min, which was kept for 4 min. Flow rates were kept at 350 nl/min. Re-equilibration was carried out for 4 μl of 0.1% buffer A at a pressure of 700 bar. Fragmentation of precursor ions was performed by higher-energy collisional dissociation with a normalized collision energy of 27%. The DIA–MS method consisted of an MS1 scan of the 350 to 1650 m/z range (AGC target of 3 × 10^6^, maximum injection time of 60 ms) at a resolution of 60,000 and 32 DIA segments (File S7, AGC target of 3 × 10^6^, maximum injection time of 45 ms). The acquisition order of samples was randomized to avoid bias.

##### Proteomics data analysis

All raw files were analyzed with Spectronaut Pulsar X (version 12.0 except for the NR experiment, which was analyzed by version 13.8) with default settings except that the “Quantification” data filtering parameter “Q value” was set to “complete” (56). Spectronaut raw data are available in File S8. DIA hybrid spectra were searched against in-house generated libraries of plasma and liver using the same LC setup searching against mouse FASTA Uniprot database (version April 2019 containing 61,000 entries). For the analysis of DIA data with Spectronaut, the precursor isotope is used. The mass tolerance for the precursor ion envelope in MS1 is determined from the data using a calibration search, with a starting width of 20 ppm (reported in the analysis summary of the Spectronaut experiment [SNE file]). The retention time tolerances are calculated dynamically along the retention time. Retention time alignment is performed per run based on the iRT system (57). For the library generation, the best six fragment ions are selected (by intensity, minimally 3, >90% will have six fragments). The mass tolerance for the main library search is determined from the data using a calibration search, with a starting width of 20 ppm (for MS1 and MS2 separately). This is reported in the analysis summary of the Spectronaut experiment (SNE file). The FDR was calculated runwise on precursor level (1%) and experiment wide on protein group level (1%). The FDR method of Storey was used (58). For comparison at individual time points and for the NR supplementation study, proteins with a significantly altered abundance were identified using the R package LIMMA (59). Significantly enriched GO terms were identified through the R package ClusterProfiler (47), using the list of detected proteins as a background reference. Dot plots with GO terms display the 10 terms with the highest gene ratio or all GO terms when fewer than 10 significant terms were detected.

#### Mitochondria isolation

Liver mitochondria were isolated from female WT and HNKO mice at 11 to 13 weeks of age, after 3 weeks of PD feeding. Mitochondria isolation and NAD^+^ measurements were performed as previously described (16). Mitochondria pellets and crushed liver from the same mice were subject to Western blot analysis as described below.

#### Western blot analyses

Approximately 20 mg of pulverized tissue or one pellet of isolated mitochondria was homogenized in 500 μl lysis buffer (for tissue) and 100 μl (for mitochondria) as previously described (41) using a Tissuelyser II (Qiagen), 2 × 30 s of 30 Hz. Lysates were incubated end over end for 45 min at 4 °C and were centrifuged for 10 min at 16,000*g* at 4 °C. Protein concentration was determined using the bicinchoninic acid assay (23227; Thermo Fisher Scientific). Electrophoresis, transfer, and immunoblotting were performed as previously described (41). Membranes were incubated according to the manufacturer's instructions with the following antibodies: acetyl-lysine (catalog no. 9441s; Cell Signaling) and malonyl-lysine (catalog no. 14942; Cell Signaling). After wash in Tris buffered saline with Tween-20, membranes were incubated with horseradish peroxidase (HRP)-conjugated anti-rabbit antibodies (170-6515; Bio-Rad). Chemiluminescence was detected as previously described (41). Band volume was normalized to the volume of internal control of mixed samples and loaded on all gels.

#### High-resolution respirometry

Oxygen consumption was measured from liver samples as previously described (16), obtained from female WT and HNKO mice. Female WT and HNKO mice were fed PD from 9 to 13 weeks of age for 3 weeks and were sacrificed as described previously. Liver biopsies were extracted and added to ice-cold buffer for high-resolution respirometry as previously described (16), using an Oxygraph-2k (Oroboros Instruments). The liver sample was weighed and added to the respirometer. Leak respiration was measured after adding 2 mM malate, 10 mM glutamate, and 5 mM pyruvate. ADP was added at increasing concentrations from 0.05 to 5 mM to assess ADP sensitivity in a physiological range. About 10 mM of the succinate was added to measure complex I+II linked respiration. Next, cytochrome c (10 μM) was added to control for outer mitochondrial membrane integrity, and finally, 0.25 μM of FCCP was added to measure the maximal uncoupled respiration. Oxygen consumption was normalized to tissue wet weight.

#### H_2_O_2_ measurement

The O2k-Fluorometer (Oroboros) was used to measure H_2_O_2_ release from the liver sample, prepared in the same way as for mitochondrial respiratory capacity. H_2_O_2_ release was measured with Amplex Red (AR), which in the presence of HRP reacts with H_2_O_2_ in a 1:1 relationship to form the stable fluorescent compound resorufin. Addition of SOD will convert released superoxide into H_2_O_2_. Resorufin production is detected as an increase in the fluorescence intensity. In liver tissue, AR is converted to resorufin by a carboxylesterase in the absence of H_2_O_2_ and HRP. This reaction can be inhibited by PMSF (60). Calibrations with H_2_O_2_ was included in the protocol to be able to convert the readout into pmol H_2_O_2_/s/mg. The following protocol was applied in MiR05 in the oxygen range from 200 to 100 nmol/ml. PMSF (100 μM), SOD (5 U/ml), and AR (20 μM) were added together, followed by HRP (1 U/ml), before the first H_2_O_2_ was added (0.1 μM). Malate (2 mM) and pyruvate (5 mM) were added before the next H_2_O_2_ calibration (0.1 μM), then succinate (1 mM) was used, and finally, the last H_2_O_2_ calibration (0.1 μM) was made. All measurements were made in duplicate.

#### Statistics

Data in bar graphs are presented as mean ± SD, except for scoring data, which are displayed as median. Nonparametric scoring data were tested using Kruskal–Wallis test. Mean ranks between groups were compared while correcting for FDR using Benjamini, Krieger, and Yekutieli two-stage step-up method. Remaining data were analyzed using two-way ANOVA with Sidak's multiple comparison post hoc test in Sigmaplot 13.0 (Systat Software Inc) and GraphPad Prism 9.1.0 (GraphPad Software). Sequencing data and proteomics were analyzed as described in the relevant sections. Data from the oxygraph measurements were analyzed with repeated two-way ANOVAs for each step. Data from all ADP concentrations were analyzed together. Data with unequal variance were transformed using log transformation. If equal variance was still not obtained, data were analyzed with Kruskal–Wallis test, with correction for FDR as described previously. All nonparametric statistical analyses were performed using GraphPad Prism 9.1.0. Pearson's correlation coefficient was calculated using GraphPad Prism 9.2.0. Statistical significance was defined as *p* < 0.05. ∗/∗∗ indicates effects of genotype, *p* < 0.05/0.01, respectively. ^#^/^##^ indicates effects of treatment (diet or NR), *p* < 0.05/0.01, respectively.

#### Study approval

All animal experiments were performed in accordance with the European directive 2010/63/European Union of the European Parliament and the Council for the protection of animals used for scientific purposes. Ethical approval was given by the Danish Animal Experiments Inspectorate (#2015-15-0201-00796).

## Data availability

The MS proteomics data have been deposited to the ProteomeXchange Consortium *via* the PRIDE (61) partner repository https://www.ebi.ac.uk/pride/archive set identifier PXD017170. The RNA-Seq datasets were uploaded to the Gene Expression Omnibus database (ID GSE144443 for the time course study, GSE173406 for the liver *versus* hepatocyte study and GSE174234 for the single cell analysis). Codes for R figure generation, RNA-Seq and proteomics analysis are available on http://www.github.com/mortendall

## Supporting information

This article contains supporting information.

## Conflict of interests

The authors declare that they have no conflicts of interest with the contents of this article.
