# Supplementary material for: Hepatocyte-specific perturbation of NAD+ biosynthetic pathways in mice induces reversible nonalcoholic steatohepatitis–like phenotypes
Source: J Biol Chem. 2021 Nov 8;297(6):101388. doi: 10.1016/j.jbc.2021.101388 (PMC8648833; doi:10.1016/j.jbc.2021.101388)
Supplement: Figures S1–S7 [file mmc1.pdf]

Supporting Figure 1

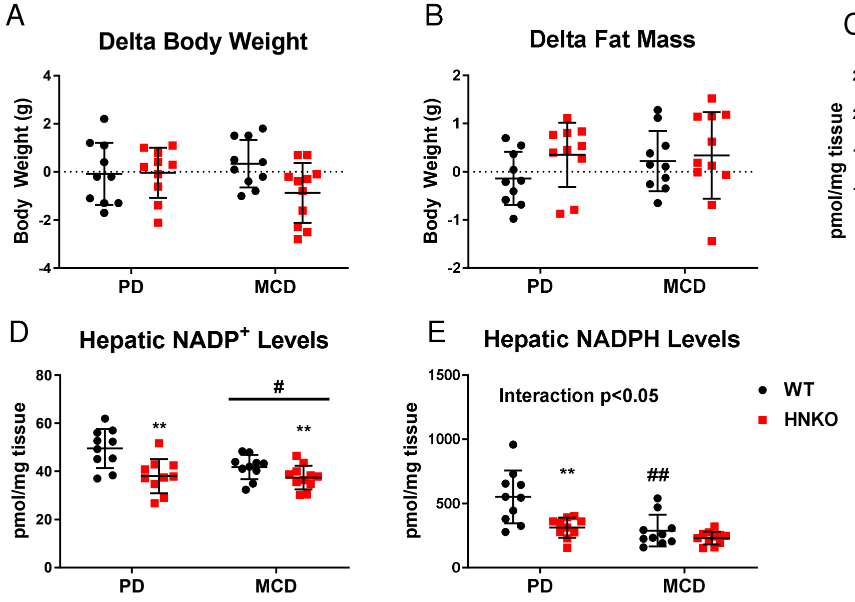

Supporting Figure 1: Supporting data for the MCD feeding- experiment. (A) delta body weight and (B) delta fat mass for WT and hepatocyte-specific Nampt knockout (HNKO) mice after 3 weeks of low-methionine, choline deficient HFD (MCD) feeding or a L-amino acid defined purified control diet (PD). (C) Hepatic NAD+, (D) NADP+, and (E) NADPH content. n= 10-11. */** indicate effects of genotype, p<0.05/0.01, respectively. #/## indicate effects of diet or time, p<0.05/0.01, respectively.

Supporting Figure 2

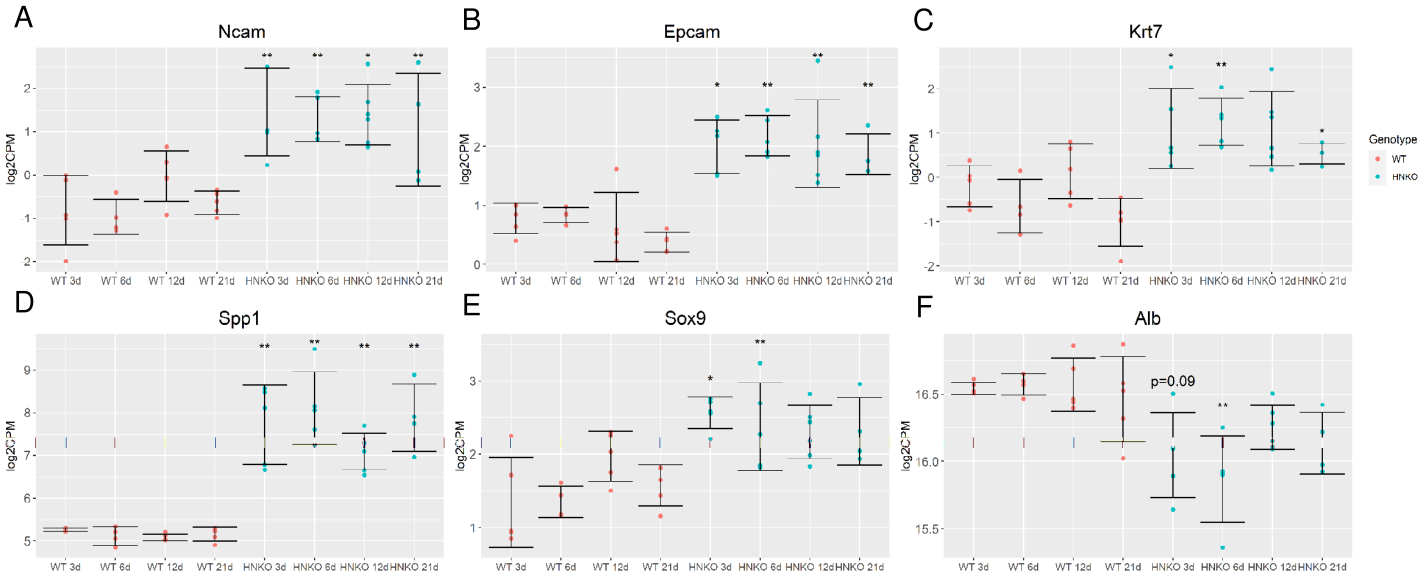

Supporting Figure 2: Supporting data for the MCD feeding- experiment. Hepatic expression of the oval cell/cholangiocyte markers (F) *Ncam1,* (G) *Epcam,* (H) *Krt7,* (I) *Spp1,* and (K) *Sox9*, and the hepatocyte marker (L) *Alb* following 3-21 days of PD feeding. n = 5-6 */** indicate effects of genotype, p<0.05/0.01, respectively.

Supporting Figure 3

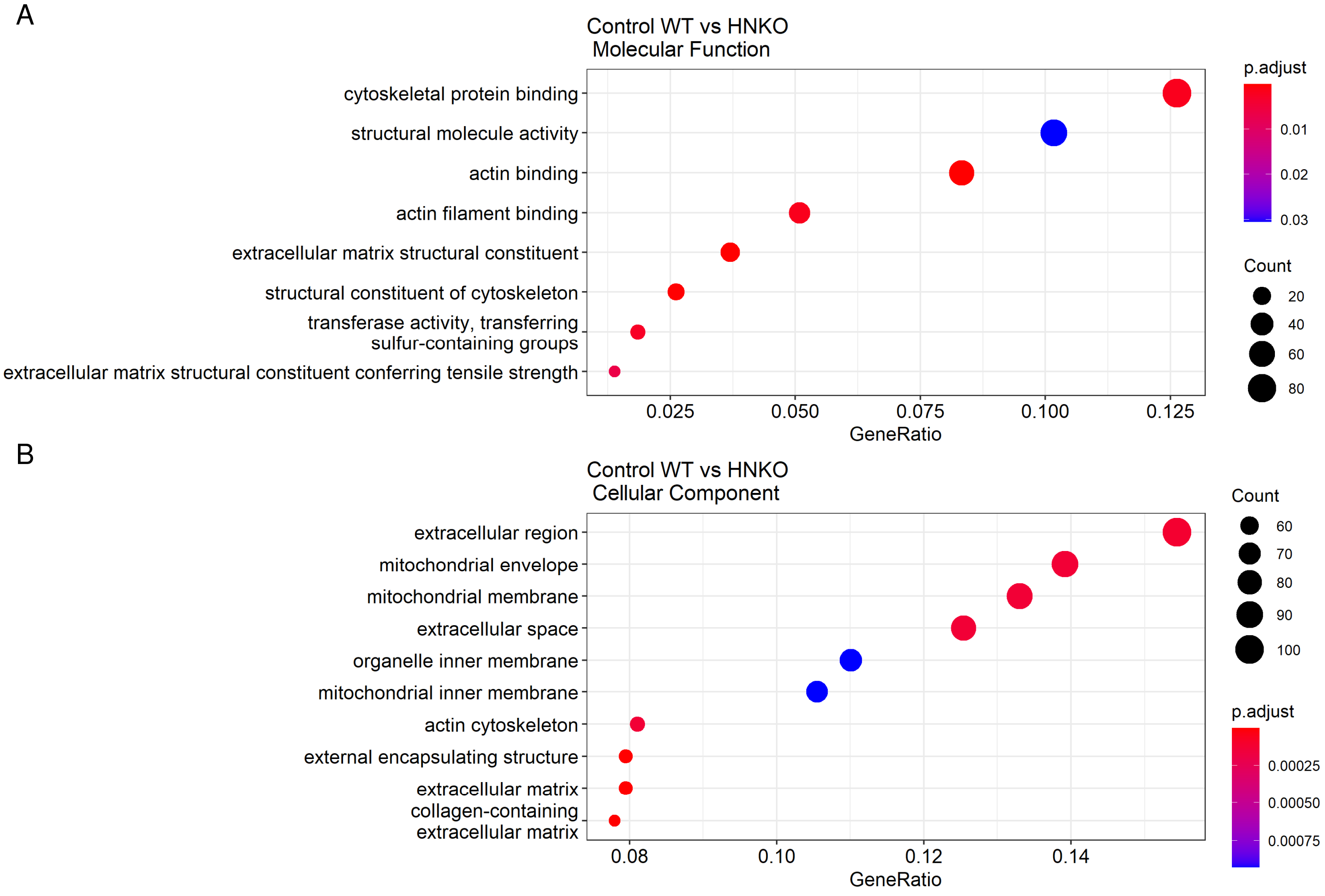

Supporting Figure 3: HNKO mice have an enrichment of proteins associated with cytoskeletal organization and collagen metabolism. Gene ontology enrichment analysis for (A) molecular function and (B) cellular component of liver proteins with a differential abundance between HNKO mice and WT mice without NR intervention. n=6-7. GO analysis was performed using the R package ClusterProfiler. n=7-10.

Supporting Figure 4

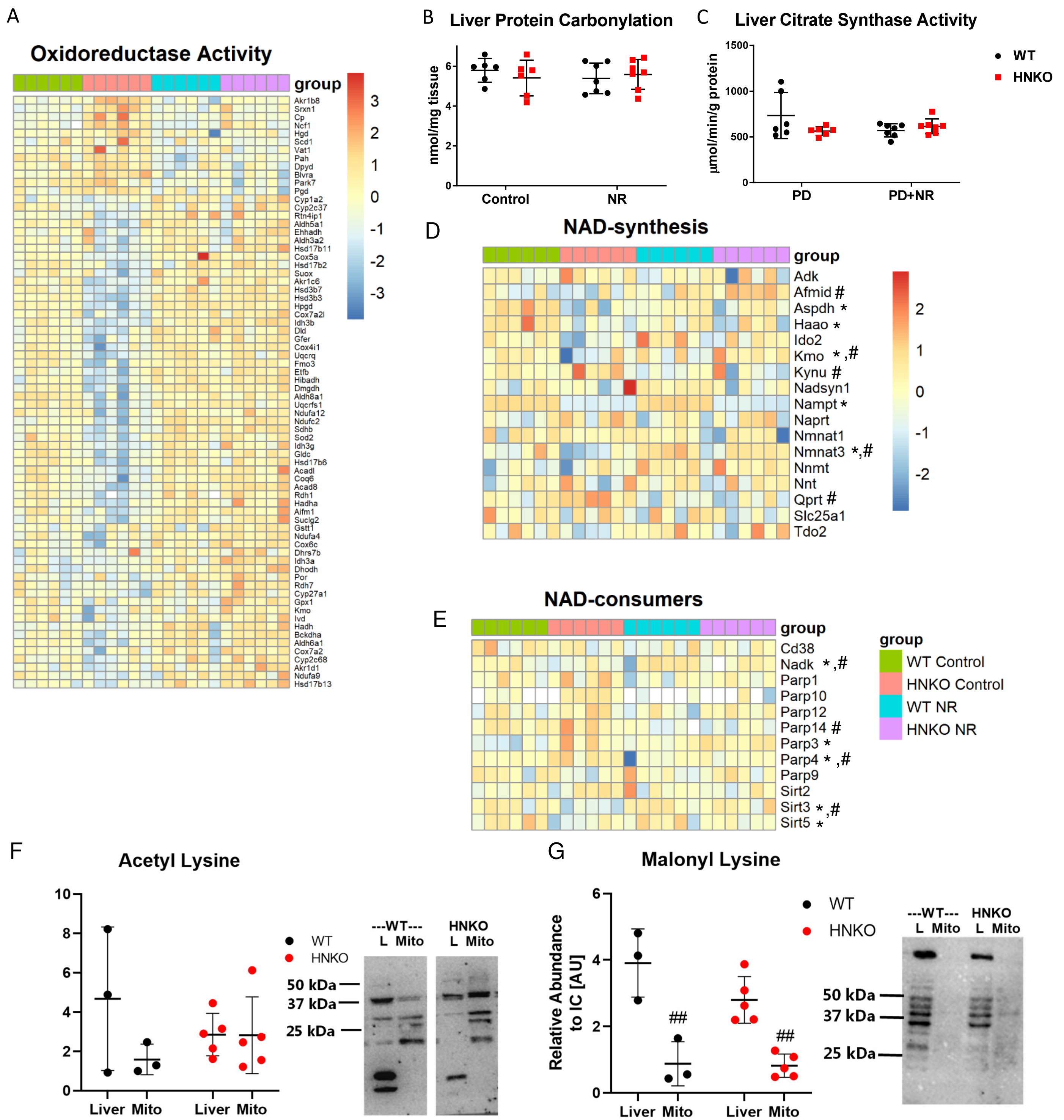

Supporting Figure 4: Supporting data for the NR prevention study. (A) Heatmap showing abundance of differentially abundant proteins for HNKO ± NR from the term *oxidoreductase activity.* (B) Liver citrate synthase activity, as a measure of total mitochondrial content. (C) Liver protein carbonylation content as a stable measure of hepatic oxidative stress. (D) selected hepatic proteins involved in NAD+ synthesis and (E) selected hepatic proteins involved in NAD+ consumption. n=5-6. Abundance of lysine (F)acetylation and (G) malonylation in full liver and mitochondria from HNKO and WT mice, n=3-5. */** indicate effects of genotype, p<0.05/0.01, respectively. #/## indicate effects of treatment (D-E) or cellular compartment (G), p<0.05/0.01, respectively.

Supporting Figure 5

A

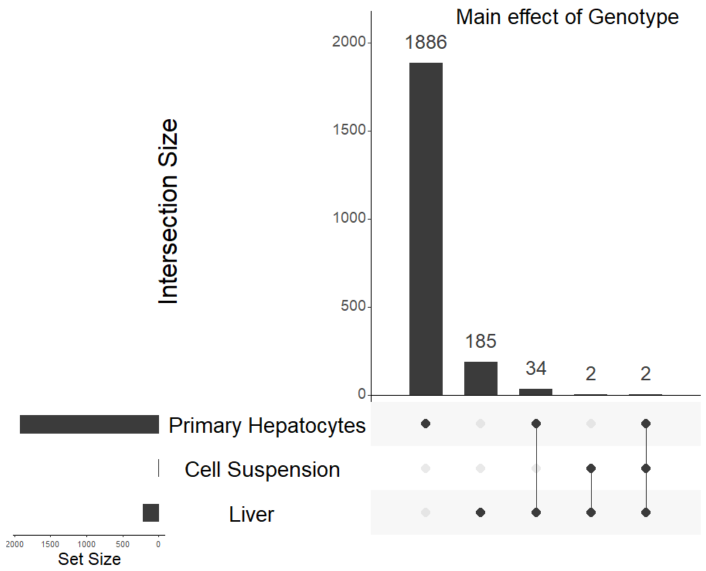

Supporting Figure 5: Supporting data from primary hepatocyte RNAseq data (A) Upset plot showing overlap for genes differentially expressed by genotype for liver (L), cell suspension prior to plating (CS) and primary hepatocytes following 24h of culturing. (PH). n=6. */** indicate effects of genotype, p<0.05/0.01, respectively. MDS plot was calculated using the R package Limma. Differential gene expression was analyzed using the R package edgeR.

Supporting Figure 6

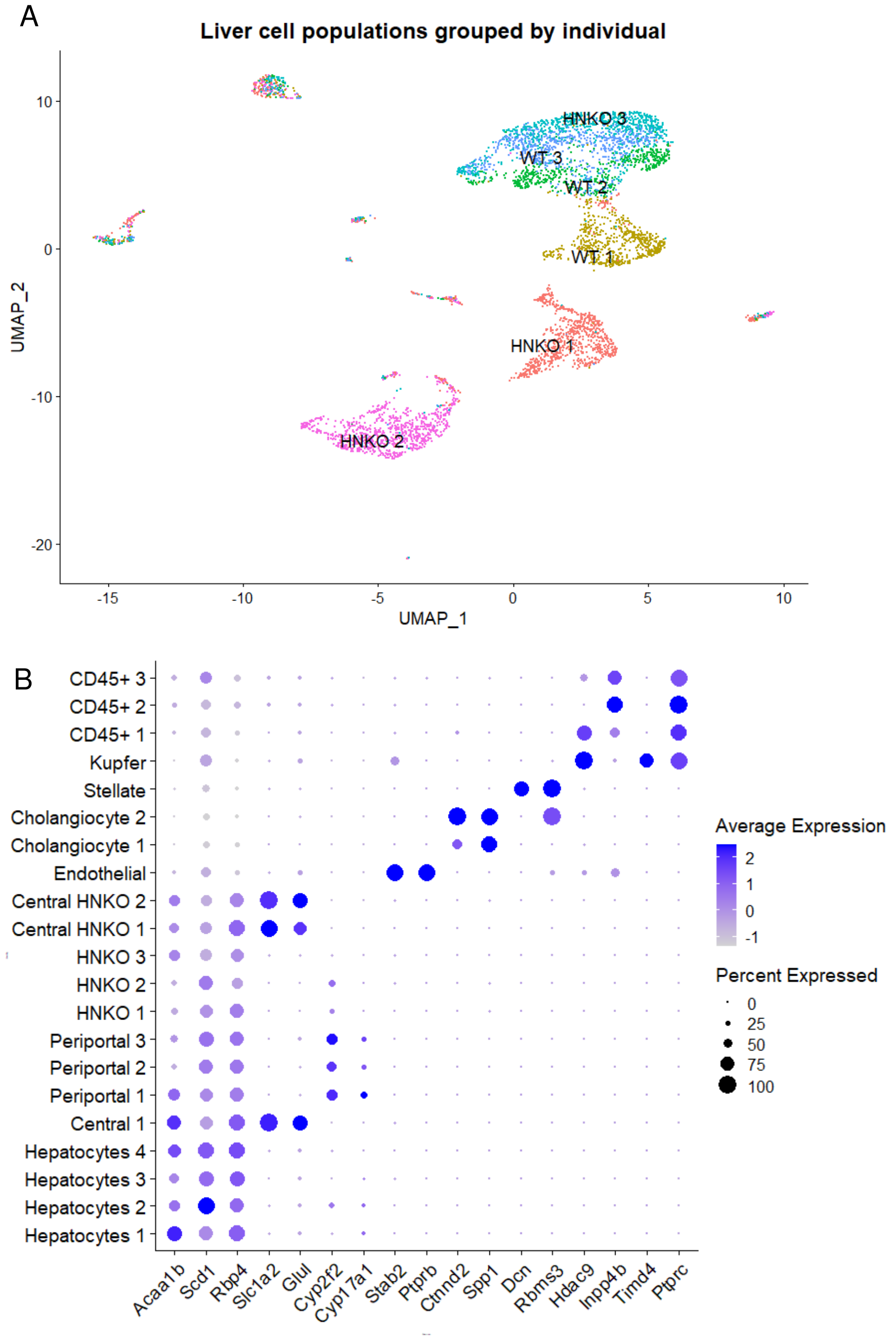

Supporting Figure 6: Single-nucleus RNAseq analysis shows that liver cells largely separate based on donor mouse. (A) UMAP plot showing separation of samples based on individual liver samples. (B) overview of markers used for cell-type annotation. n=3. Clusters were identified using the R package Seurat. Differentially expressed genes were identified using the R package Seurat, using the FindMarkers function on sequencing data from the hepatocyte subset, using genotype as identity classes.

Supporting Figure 7

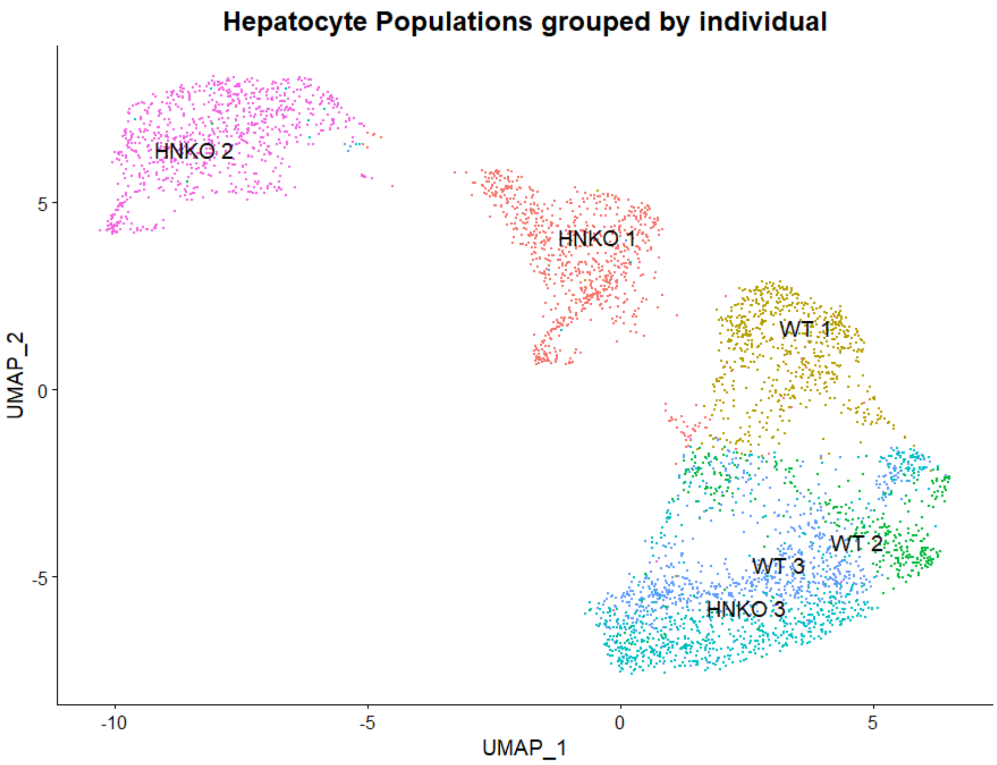

B

	Fibrosis	Portal inflam	Necrosis
HNKO 1	1	1	2
HNKO 2	0.5	0.5	2
HNKO 3	1	0.5	0
WT 1	0	0	0
WT 2	0	0	0
WT 3	0	0	0

C

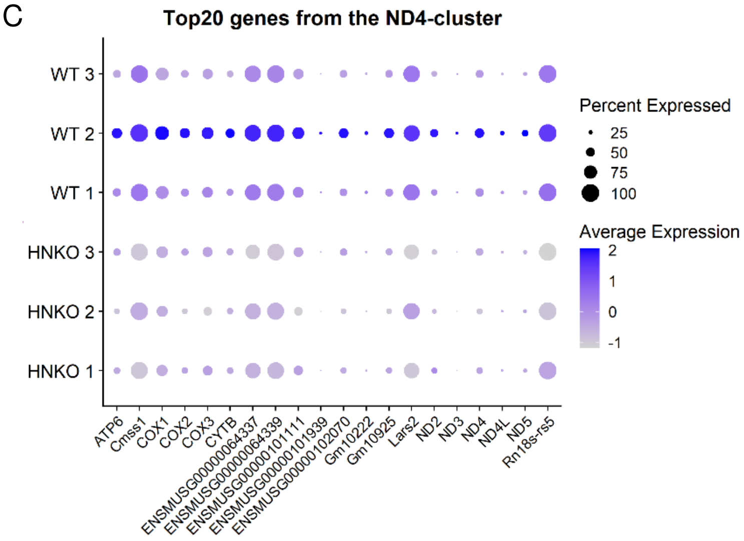

D

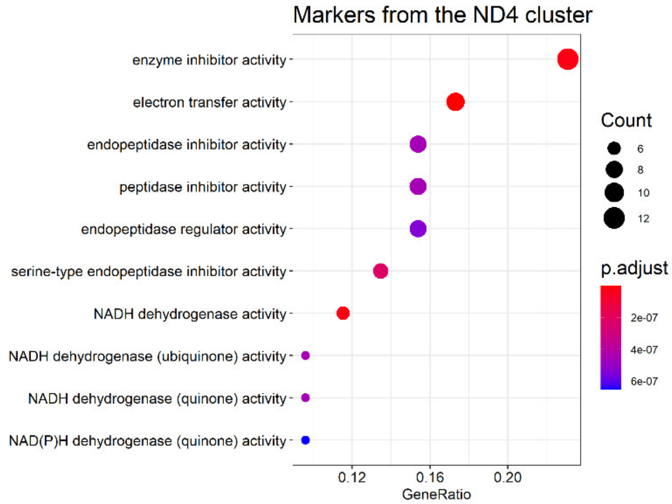

Supporting Figure 7: Analysis of hepatocyte sub-populations confirms that hepatocytes separate according to donor mouse. (A) UMAP plot showing separation of hepatocyte sub-populations, colored for individual liver samples. (B) Table showing individual histology scores for mice included in the scRNAseq analysis. (C) Dotplot showing abundance of the 20 most significantly enriched genes from the ND4 cluster, (D) Gene ontoloty enrichment of markers from the ND4 cluster.
